# Explainable machine learning to predict immunotherapy outcomes in metastatic renal cell carcinoma - Meet-URO 15-AI study

**DOI:** 10.1038/s41698-026-01576-x

**Published:** 2026-07-02

**Authors:** Sara Elena Rebuzzi, Vanja Miskovic, Giuseppe Fornarini, Sara Ferri, Sebastiano Buti, Matteo Piceni, Alessio Signori, Leonardo Provenzano, Giuseppe Luigi Banna, Pasquale Rescigno, Marco Maruzzo, Davide Bimbatti, Beatrice Ramella Pollone, Umberto Basso, Ugo De Giorgi, Paolo Pedrazzoli, Luca Galli, Paolo Andrea Zucali, Fabrizio Di Costanzo, Aruni Ghose, Alessandra Laura Giulia Pedrocchi, Arsela Prelaj

**Affiliations:** 1 Medical Oncology Unit, Ospedale San Paolo, Savona, Italy; 2 Department of Electronics, Information and Bioengineering, Polytechnic University of Milan, Milan, Italy; 3 Department of Medical Oncology, Fondazione IRCCS Istituto Nazionale dei Tumori (INT), Milan, Italy; 4 Medical Oncology Unit, IRCCS Azienda Ospedaliera Metropolitana (IRCCS AOM), IRCCS Ospedale Policlinico San Martino, Genova, Italy; 5 Medical Oncology Unit, University Hospital of Parma, Parma, Italy; 6 Department of Medicine and Surgery, University of Parma, Parma, Italy; 7 Department of Health Sciences (DISSAL), Section of Biostatistics, University of Genova, Genova, Italy; 8 Department of Oncology, Portsmouth Hospitals University NHS Trust, Portsmouth, UK; 9 Faculty of Science and Health, School of Pharmacy and Biomedical Sciences, University of Portsmouth, Portsmouth, UK; 10 Translational and Clinical Research Institute, Centre for Cancer, Newcastle University, Newcastle Upon Tyne, UK; 11 Interdisciplinary Group for Translational Research and Clinical Trials, Urological Cancers (GIRT-Uro), Candiolo Cancer Institute-FPO-IRCCS, Candiolo, Turin Italy; 12 Oncology Unit 3, Istituto Oncologico Veneto-IOV-IRCCS, Padua, Italy; 13 Oncology Unit 1, Istituto Oncologico Veneto-IOV-IRCCS, Padua, Italy; 14 Department of Medical Oncology, IRCCS Istituto Romagnolo per lo Studio dei Tumori (IRST) “Dino Amadori”, Meldola, Italy; 15 Medical Oncology Unit, Fondazione IRCCS Policlinico San Matteo, Pavia, Italy; 16 Medical Oncology Unit 2, Azienda Ospedaliera Universitaria Pisana, Pisa, Italy; 17 Department of Biomedical Sciences, Humanitas University, Pieve Emanuele, Italy; 18 Division of Oncology, IRCCS Humanitas Research Hospital, Milan, Italy; 19 Medical Oncology, University of Naples Federico II, Naples, Italy; 20 Barts Cancer Centre, St Bartholomew’s Hospital, Barts Health NHS Trust, London, UK

**Keywords:** Biomarkers, Cancer, Computational biology and bioinformatics, Immunology, Oncology

## Abstract

Metastatic renal cell carcinoma remains clinically challenging because of heterogeneous outcomes and limited predictive biomarkers for immunotherapy. We performed an explainable machine learning analysis using data from the multicenter retrospective Meet-URO 15 study, including 571 patients with metastatic renal cell carcinoma treated with second-line or later nivolumab. Clinical and inflammatory variables were used to develop classification models for disease control rate, progression-free survival at 3 and 9 months, and overall survival at 6, 18 and 24 months, as well as survival models for continuous progression-free and overall survival. Model performance was assessed using weighted F1-score for classification and concordance index for survival analysis, with interpretability provided through Shapley additive explanations. The best classification performance was observed for 6-month overall survival using a support vector machine model combined with minimum redundancy maximum relevance feature selection, achieving an F1-score of 0.81 on the test set and 0.77 in external validation. In survival analysis, random survival forest achieved a test-set concordance index of 0.68 for overall survival. Inflammatory indices, IMDC score, hemoglobin, lymphocytes and platelets consistently emerged as relevant prognostic features. These findings support explainable machine learning as a transparent approach to refine outcome prediction in immunotherapy-treated metastatic renal cell carcinoma.

**Figure:**
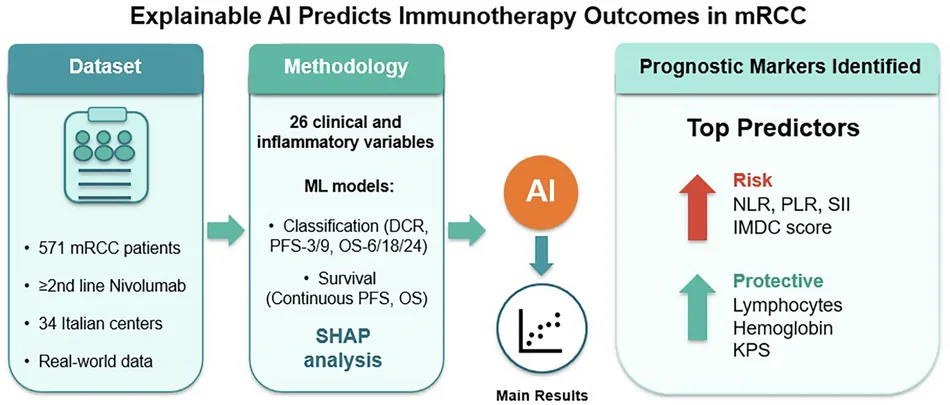

## Introduction

The treatment landscape of metastatic renal cell carcinoma (mRCC) has profoundly changed over the last decade, with the availability of several therapeutic strategies that allow treatment to be tailored to individual patients. These include cytoreductive surgery, targeted therapies such as multitarget tyrosine kinase inhibitors (TKIs) and mammalian target of rapamycin (mTOR) inhibitors, as well as immune checkpoint inhibitors administered either as monotherapy or in combination regimens^1–3^. However, mRCC is still a significant clinical challenge due to its heterogeneous molecular nature, with highly variable clinical outcomes and response to therapy^4^. In addition, the absence of reliable prognostic biomarkers complicates treatment selection and risk stratification^5^.

Traditional prognostic models, such as the International Metastatic RCC Database Consortium (IMDC) score, relied on clinical and laboratory variables and were developed during the era of VEGF-targeted therapies and may not fully capture the nuances of immunotherapy response^5,6^. Indeed, immunotherapy efficacy is governed by a complex interplay of biological factors, including tumor mutational burden, immune cell infiltration patterns, PD-L1 expression, and dynamic tumor-immune microenvironment interactions, that are not reflected in conventional clinical or laboratory parameters. Moreover, the response to immune checkpoint inhibitors is inherently non-linear and patient-specific, driven by multidimensional interactions that traditional regression-based statistical models are structurally ill-equipped to model. For this reason, many studies have been conducted to develop updated clinical prognostic models^6,7^ or to validate prognostic scores in mRCC patients receiving immunotherapy^8–10^.

In 2021, the Meet-URO 15 study enrolled 571 mRCC patients receiving ≥2^nd^ line nivolumab and developed a novel prognostic score (Meet-URO score), which showed higher prognostic stratification compared with the standard of care IMDC score^6^.

Since cancer heterogeneity and immune system complexity do not allow to identify a single binary biomarker, artificial intelligence (AI) can help integrate multimodal data and identify composite prognostic and/or predictive models through Machine Learning (ML) and Deep Learning (DL) methodologies^11^. For this reason, AI has emerged as an innovative tool in oncology, offering enhanced capabilities in diagnosis, prognostication and treatment planning^12^.

In RCC, AI applications have demonstrated promising results in analyzing and integrating histopathological, genomic, clinical and imaging data for more precise tumor subtyping and prognostication to refine RCC management^12–14^. The use of AI for immunotherapy efficacy prediction in cancer patients is an emerging and promising area of research^15^, with its most relevant applications in mRCC focused on radiomic features as clinical predictors of immunotherapy response^12,16^. However, the application of AI to identify prognostic factors in mRCC patients receiving immunotherapy remains poorly explored and the few analyses conducted have shown AI-driven models reaching robust prognostic capabilities^12,17–20^. Specifically, existing studies in this field are affected by limitations that reduce their generalizability and clinical applicability: some were conducted on single-center cohorts of limited size, restricting the external validity of their findings^18^; several relied on histological or genomic data that require dedicated tissue analyses and are therefore difficult to access in routine clinical practice^13^; and few provided interpretable models, that identify which clinical variables most meaningfully drive the predicted outcome.

In this context, we have conducted an AI-driven analysis applying state-of-the-art ML techniques to identify the most important prognostic factors influencing the immunotherapy efficacy in a large-scale database on mRCC patients receiving nivolumab from the Meet-URO 15 study.

## Results

### Dataset characteristics and preparation

The dataset comprised 571 mRCC patients enrolled in the Meet-URO 15 study. The data was split into training, test and external validation sets at fixed ratios of 70%-20%-10%, respectively, consistently across all analyses. However, the number of patients effectively included in each set varied across ML tasks and outcomes, as patients lacking outcome information for a specific endpoint were excluded from the corresponding analysis. As a result, the effective sample size differed across tasks, with the continuous OS outcome being the only one for which all 571 patients could be included. The descriptive analysis of the full cohort is reported in Table 1, while the number of patients included in each set for each specific outcome is detailed in Supplementary Table 1. Analysis of missingness patterns revealed that missing values were predominantly dependent on the center of data collection rather than on the missing values themselves, supporting the Missing At Random (MAR) assumption conditionally on the center, as visually confirmed in Supplementary Fig. 1.

**Table 1:** Descriptive analysis for training, testing and external validation sets

		Train set (*n* = 400)	Test set (*n* = 114)	External Validation set (*n* = 57)
Patients’ characteristics	Age (years)	Median = 63 (Q1–Q3: 52–70) Missing: 2 (0.5%)	Median = 62 (Q1–Q3: 54–69) Missing: 0 (0%)	Median = 63 (Q1–Q3: 52–71) Missing: 0 (0%)
	Sex	Male: 280 (70%) Female: 120 (30%)	Male: 84 (74%) Female: 30 (26%)	Male: 38 (67%) Female: 19 (33%)
	KPS	<80%: 61 (15%) ≥80%: 335 (84%) Missing: 4 (1%)	<80%: 19 (17%) ≥80%: 94 (82%) Missing: 1 (1%)	<80%: 8 (14%) ≥80%: 49 (86%) Missing: 0 (0%)
Tumor characteristics	Histology	1: 339 (85%) 2: 28 (7%) 3: 10 (2%) 4: 20 (5%) Missing: 3 (1%)	1: 95 (83%) 2: 10 (9%) 3: 2 (2%) 4: 6 (5%) Missing: 1 (1%)	1: 44 (77%) 2: 4 (7%) 3: 5 (9%) 4: 4 (7%) Missing: 0 (0%)
	IMDC score at diagnosis	F: 105 (26%) I: 211 (53%) P: 28 (7%) Missing: 56 (14%)	F: 32 (28%) I: 57 (50%) P: 8 (7%) Missing: 17 (15%)	F: 28 (49%) I: 25 (44%) P: 4 (7%) Missing: 0 (0%)
	Bone metastases	No: 250 (62%) Yes: 150 (38%)	No: 76 (67%) Yes: 38 (33%)	No: 42 (74%) Yes: 15 (26%)
Therapy characteristics	Line of immunotherapy	2: 274 (68%) 3: 83 (21%) ≥4: 43 (11%)	2: 73 (64%) 3: 28 (25%) ≥4: 13 (11%)	2: 47 (82%) 3: 9 (16%) ≥4: 1 (2%)
Laboratory data at baseline	Hemoglobin (g/dL)	Median = 12.1 (Q1–Q3: 10.9–13.4) Missing: 114 (29%)	Median = 11.9 (Q1–Q3: 10.3–13.4) Missing: 32 (28%)	Median = 11.7 (Q1–Q3: 10.5–13.2) Missing: 0 (0%)
	Lymphocytes (cells/mm³)	Median = 1400 (Q1–Q3: 1070–1800) Missing: 75 (19%)	Median = 1390 (Q1–Q3: 1018–1895) Missing: 24 (21%)	Median = 1330 (Q1–Q3: 1090–1780) Missing: 0 (0%)
	Eosinophils (cells/mm³)	Median = 110 (Q1–Q3: 80–200) Missing: 91 (23%)	Median = 110 (Q1–Q3: 90–218) Missing: 28 (25%)	Median = 50 (Q1–Q3: 15–120) Missing: 0 (0%)
	Platelets (cells/mm³)	Median = 230500 (Q1–Q3: 187000–309000) Missing: 30 (8%)	Median = 251000 (Q1–Q3: 197750–310250) Missing: 10 (9%)	Median = 234000 (Q1–Q3: 176000–332000) Missing: 0 (0%)
	NLR *(after imputation)*	Median = 2.91(Q1–Q3: 2.00–4.24)	Median = 2.92 (Q1–Q3: 2.24–4.15)	Median = 2.32 (Q1–Q3: 1.58–4.11)
	PLR *(after imputation)*	Median = 172 (Q1–Q3: 122–249)	Median = 172 (Q1–Q3: 137–265)	Median = 167 (Q1–Q3: 116–257)
	SII *(after imputation)*	Median = 747,136 (Q1–Q3: 421,315–1,116,143)	Median = 748,819 (Q1–Q3: 570,690–1,019,858)	Median = 600,650 (Q1–Q3: 329,000–941,520)
Survival information	OS (months)	Median 26 (95% CI: 20 – 32)	Median 25 (95% CI: 21 – 42)	Median 21 (95% CI: 11 – 32)

Q1 first quartile; Q3 third quartile; KPS Karnofsky performance status, IMDC International Metastatic RCC Database Consortium, NLR neutrophils-to-lymphocytes ratio, PLR platelets-to-lymphocytes ratio, SII systemic immune-inflammation index; OS overall survival.

For IMDC score (encoding for the analysis): F = favorable-risk (1), I = intermediate-risk (2), P = poor-risk (3).

For histology: 1 = clear cell, 2 = papillary, 3 = chromophobe, 4 = other.

### Feature selection

Following a hypothesis-driven selection process, where variables were selected based on their prognostic significance in previous literature and expert clinical input, 26 clinical features were retained for the analysis (Supplementary Table 2).

Most of the data were collected at baseline of immunotherapy, except for two prognostic scores (IMDC score and MSKCC score), which were taken both before the first-line therapy with an antiangiogenic tyrosine kinase inhibitor (TKI) and at baseline of immunotherapy.

Lactate dehydrogenase (LDH), despite its clinical relevance, was excluded from the analysis due to an excessive proportion of missing values (39% in the training set). Imputing such a high fraction of values would have introduced substantial uncertainty, compromising the reliability of the imputed data and potentially biasing the model’s learning process. Following imputation, the inflammatory features NLR, PLR, SII were computed and added to the feature set.

Other two features, MSKCC score at diagnosis and baseline, were eliminated due to high correlation (> 80%) with the IMDC score at diagnosis and baseline to reduce multicollinearity (Supplementary Fig. 2) and because LDH (one of the factors of MSKCC score) was missing in 45% of patients. Inflammatory indices SII, NLR and PLR were also correlated but were kept due to their clinical relevance.

Task-specific feature selection methods (MRMR, FFS, BFS, RFE for classification and univariate feature selection for survival analysis) were then applied and evaluated by incorporating them into the training pipeline, where feature selection and hyperparameter tuning were performed jointly and optimized simultaneously via cross-validation.

### ML classification analysis

DCR, PFS-9 and OS-18 results are reported in the Supplementary section, with Supplementary Figs. 3–6.

The full list of hyperparameters explored for each classification model is reported in Supplementary Table 3.

### Progression-Free Survival at 3 months (PFS-3)

The best model for PFS-3 was RF with no feature selection (RF-none) with 23 features, achieving F1 = 0.79, ACC = 0.68, AUC = 0.82 on the test set, with partially preserved performance in external validation (F1 = 0.81, ACC = 0.66, AUC = 0.62) (Supplementary Fig. 7).

**Fig. 1:**
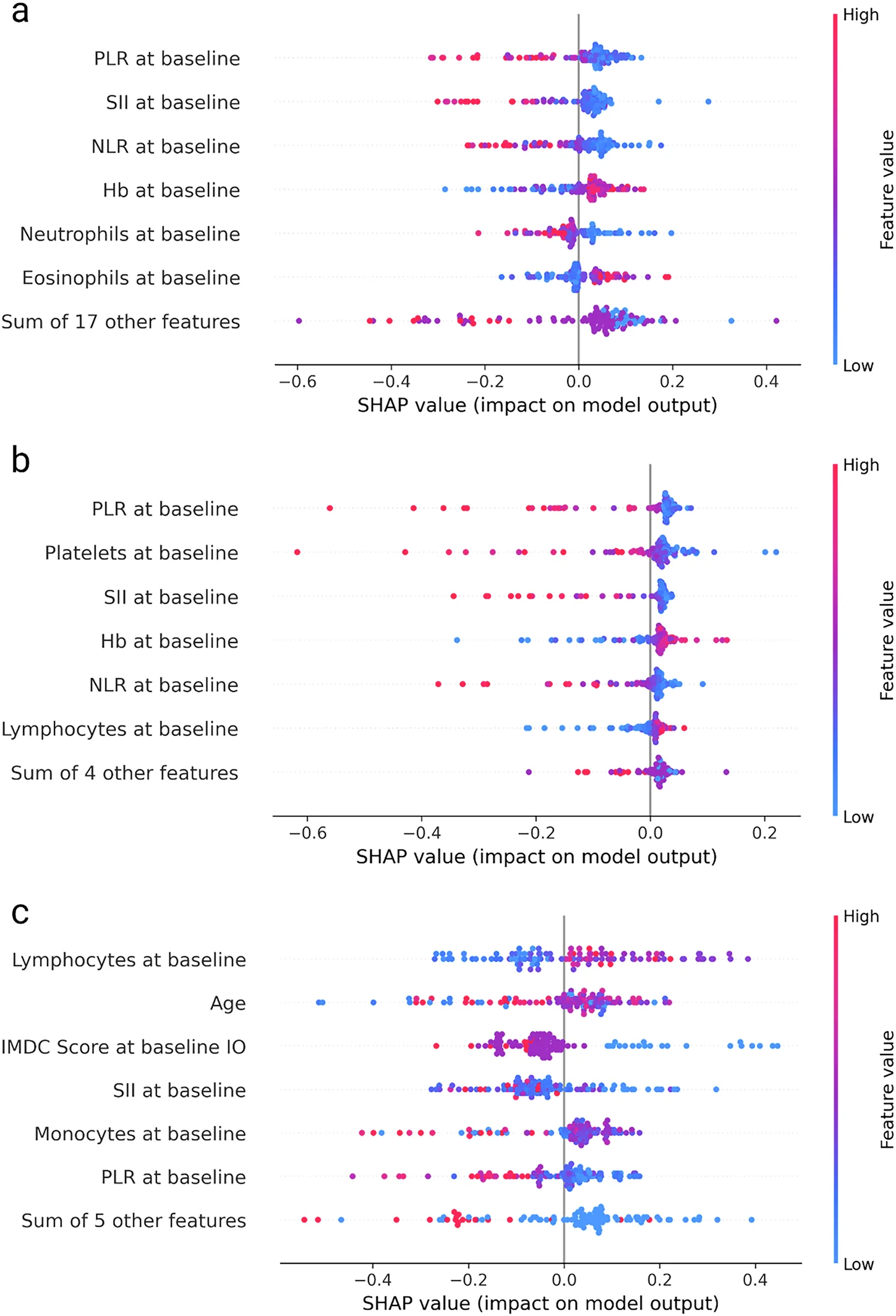
SHAP-based interpretation of the best-performing classification models. SHAP beeswarm plots for the test-set population of the best-performing classification models: **a** RF-none for PFS-3; **b** SVM-MRMR for OS-6; **c** XGB-RFE for OS-24. Each point represents an individual patient. The x-axis shows the SHAP value, indicating the impact of each feature on the model output. Color indicates the feature value, with blue representing low values and pink representing high values.

The most predictive features for PFS-3 were the three inflammatory indices NLR, PLR and SII, whose high values were associated with a shorter PFS ( < 3 months). On the contrary, high levels of hemoglobin, Neutrophils, and Eosinophils were linked to the prediction of longer PFS ( ≥ 3 months) (Fig. 1a).

### Overall Survival at 6 months (OS-6)

The SVM model trained with MRMR (SVM-MRMR), using 10 features, achieved the best performance for OS-6 prediction. The selected features included inflammatory indexes, hemoglobin levels, platelets and lymphocytes, IMDC score at baseline.

The model attained an F1-score of 0.81, ACC of 0.67, and AUC of 0.74 on the test set. On external validation, it maintained good performance, with F1 = 0.77, ACC = 0.60 and AUC = 0.66. (Supplementary Fig. 8).

The SHAP beeswarm analysis showed that higher PLR, NLR, SII were associated with an increased predicted probability of mortality within 6 months. Conversely, patients with low values of platelets and high levels of hemoglobin and lymphocytes were linked to a higher predicted likelihood of survival beyond 6 months (Fig. 1b).

### Overall Survival at 24 months (OS-24)

The best performing model for OS-24 was XGB with RFE feature selection (XGB-RFE), utilizing 11 features. This model achieved limited predictive performance on the test set (F1 = 0.60, ACC = 0.60, AUC = 0.68). On the external validation set, the model showed improved performance across all metrics (F1 = 0.68, ACC = 0.68, AUC = 0.74) (Supplementary Fig. 9).

Top predictors included lymphocytes and monocytes levels, age, IMDC score at baseline, SII and PLR.

SHAP interpretability revealed consistent significance of high inflammatory indices as negative predictors of OS-24; on the other hand, high levels of lymphocytes and low value of IMDC score at baseline were consistently associated with improved long-term survival (Fig. 1c).

### ML classification tasks summary

Table 2 summarizes the classification model performance across all clinical outcomes. Across the classification tasks, the most recurrently selected features were NLR, PLR, SII, IMDC score at baseline, lymphocyte count, and hemoglobin levels (Table 3). The number of selected features for every ML model and the confusion matrices of the best models for each task are shown in Supplementary Table 4 and Supplementary Fig. 10, respectively.

**Table 2:** Classification model performance summary across all clinical outcomes for the best models

Outcome	Model	Feature Selection	*N* Features	F1 (Test)	ACC (Test)	AUC (Test)	F1 (External validation)	ACC (External validation)	AUC (External validation)
**DCR**	ET	BFS	22	0.64	0.61	0.65	0.59	0.56	0.63
**PFS-3**	RF	none	23	0.79	0.68	0.82	0.81	0.66	0.62
**PFS-9**	ET	RFE	15	0.63	0.63	0.68	0.67	0.66	0.69
**OS-6**	SVM	MRMR	10	0.81	0.67	0.74	0.77	0.60	0.66
**OS-18**	ET	BFS	19	0.74	0.72	0.79	0.56	0.56	0.69
**OS-24**	XGB	RFE	11	0.60	0.60	0.68	0.68	0.68	0.74

DCR disease control rate; PFS-3 progression-free survival at 3 months; PFS-9 progression-free survival at 9 months; OS-6 overall survival at 6 months; OS-18 overall survival at 18 months; OS-24 overall survival at 24 months; ET Extra Trees; RF Random Forest; SVM Support Vector Machine; XGB XGBoost; RFE Recursive Feature Elimination; BFS Backward Feature Selection; FFS Forward Feature Selection; MRMR Minimum Redundancy Maximum Relevance; N number; ACC balanced accuracy; AUC Area Under the Curve.

**Table 3:** Most important features for classification models

Endpoint	Model	F1-score test set	Most relevant features								
**DCR**	ET-BFS	0.64	NLR		SII	-			baseline IMDC	KPS	Bone mts, M + , Neph
**PFS-3**	RF-none	0.79	NLR	PLR	SII	Hb	Lym	Eos	baseline IMDC	-	Neutrophils
**PFS-9**	ET-RFE	0.63	-	PLR	-	Hb	Lym	Eos	baseline IMDC	-	Sex, Diagnosis IMDC
**OS-6**	SVM-MRMR	0.81	NLR	PLR	SII	Hb	Lym	-	baseline IMDC	-	Platelets
**OS-18**	ET-BFS	0.74	NLR	PLR	-	Hb	-	-	baseline IMDC	KPS	Bone mts, Lymph node mts
**OS-24**	XGB-RFE	0.60	-	PLR	SII	-	Lym	-	baseline IMDC	KPS	Monocytes, Age

DCR disease control rate, PFS-3 progression-free survival at 3 months; PFS-9 progression-free survival at 9 months; OS-6 overall survival at 6 months; OS-18 overall survival at 18 months; OS-24 overall survival at 24 months; NLR neutrophils-to-lymphocytes ratio; PLR platelets-to-lymphocytes ratio; SII systemic immune-inflammation index; Hb hemoglobin; Lym lymphocytes; Eos eosinophils; IMDC International Metastatic RCC Database Consortium; KPS Karnofsky performance status; M+ metastatic at diagnosis; neph nephrectomy; mts metastases.

### Survival model performance for long-term outcome prediction

The hyperparameter search space for each survival analysis model is detailed in Supplementary Table 5.

In the survival analysis task, the performance of models trained on the complete set of clinical features was compared with those trained on features selected through a univariate filtering approach (Supplementary Table 6). Models developed using univariate feature selection—based on a c-index threshold of 0.55—demonstrated comparable predictive performance relative to those trained on all available features, for both OS and PFS outcomes.

The application of this feature selection method resulted in more compact models while maintaining prognostic accuracy, as evidenced by stable c-index values across both outcomes. These findings suggest that univariate selection effectively retains variables with independent prognostic value, reducing dimensionality without compromising model performance, and thereby facilitating model interpretability in the context of long-term outcome prediction.

Among the six different survival models evaluated, the best-performing survival model for OS was the RSF model applied to univariately-selected features, which achieved a c-index of 0.69 ± 0.05 on cross validation and 0.68 on the test set (Supplementary Table 6). For PFS, the best model employing univariately-selected features was SSVM, reaching c-index of 0.61 ± 0.05 and 0.66 on cross validation and test set, respectively.

### Selected prognostic features

Following the univariate screening, for OS prediction, selected features included IMDC score at diagnosis and baseline, KPS, hemoglobin at baseline, bone metastases, NLR, PLR, SII, and lymphocyte count; for PFS, selected features were: IMDC score at diagnosis and baseline, KPS, SII, PLR, Lymphocytes and Bone metastases.

### Model explainability

**Fig. 2:**
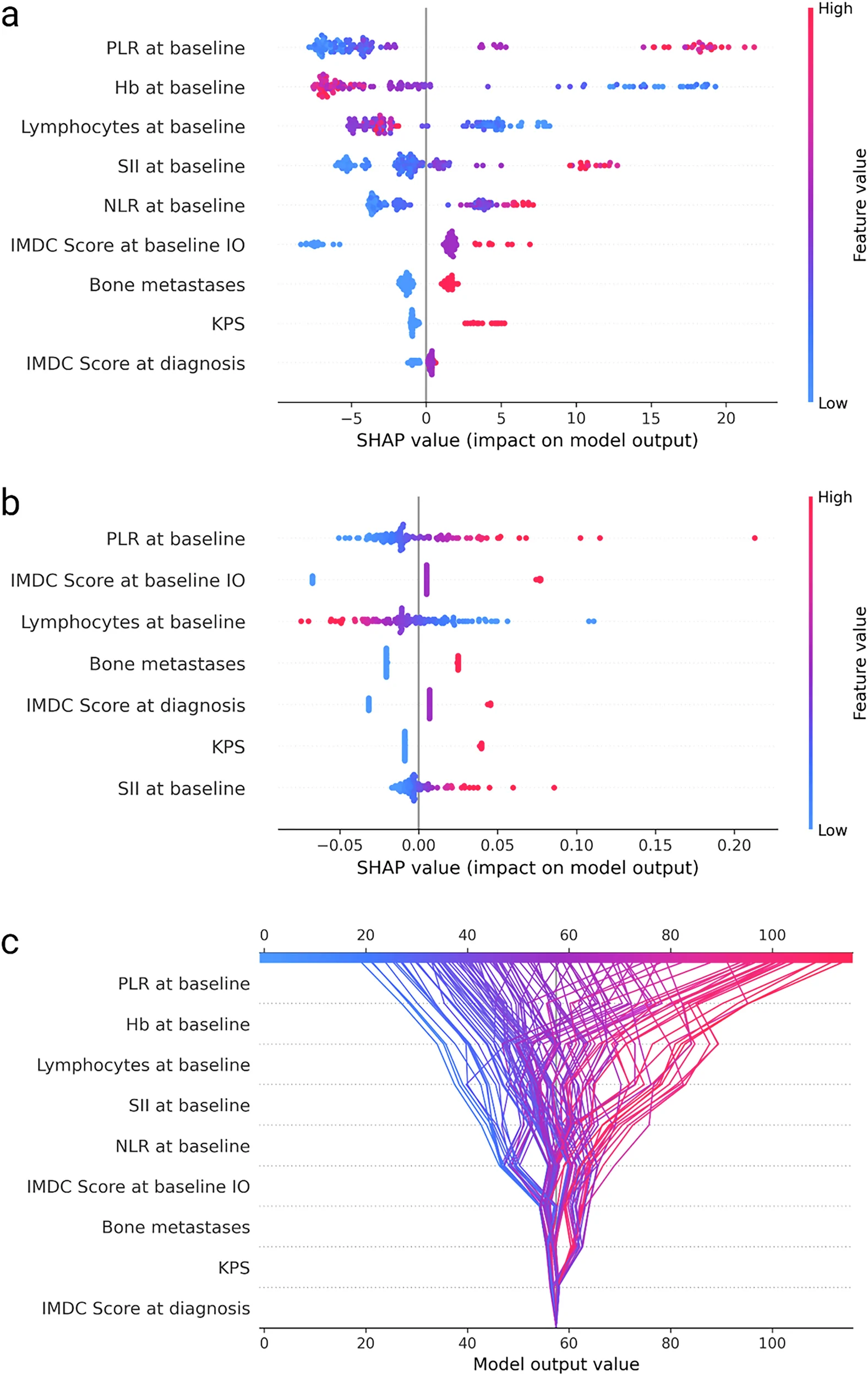
SHAP-based interpretation of survival models. SHAP graphs of survival models applied to the test set: **a** beeswarm plot of RSF-univariate for OS; **b** beeswarm plot of SSVM-univariate for PFS; **c** decision plot of RSF-univariate for OS, with features ordered by importance. In the beeswarm plots, each point represents an individual patient and color indicates the feature value, with blue representing low values and pink representing high values. In the decision plot, each line represents an individual patient and shows how feature contributions combine to determine the final model output.

The SHAP Beeswarm plot of RSF-univariate for OS (Fig. 2a**)** reveals that high values of inflammatory markers, NLR, PLR and SII, and presence of bone metastases were among the strongest features associated with a higher predicted risk of mortality. Conversely, high hemoglobin levels, KPS, lymphocyte counts and lower IMDC score were associated with favorable survival outcomes.

Complementing the RSF-based insights, SHAP analysis was also applied to the EST model trained on the full set of features.

The EST model on all features showed similar performance for OS (c-index: 0.69 ± 0.06 on cross validation and 0.68 on the test set; Supplementary Fig. 11a), demonstrating that the same features, particularly KPS, bone metastases, inflammatory markers, IMDC score and hemoglobin, remain dominant predictors, underscoring the robustness of these variables across modeling techniques.

SSVM model for PFS further confirms the relevance of PLR, IMDC score, lymphocytes, bone metastases and KPS, aligning with the key features identified for OS (Fig. 2b).

The EST model applied to PFS prediction showed comparable performance with a c-index of 0.62 ± 0.05 on cross validation and 0.65 on test set (Supplementary Fig. 11b), reflecting moderate but consistent predictive capabilities across different endpoints and modeling strategies.

The consistency in feature importance across OS and PFS tasks highlights the central role of systemic inflammation in predicting long-term outcomes in mRCC patients treated with immunotherapy.

### Decision plots: feature influence on risk stratification

The decision plots visualize how the model assigns risk over the patient population, illustrating, for instance, how features shift the model’s output towards higher or lower risk scores.

Some plots are sorted by feature importance (Fig. 2c), while others are organized via hierarchical clustering to group patients with similar prediction patterns, making outliers easier to spot (Supplementary Fig. 12a). Other plots (Supplementary Fig. 12c) highlight how clustering and feature value color gradients reveal not only the direction but also the strength of a feature’s effect across different patient risk profiles.

### Dependence plots: threshold insights for OS prediction

To further show the effect that a single feature has on the predictions made by the model, illustrating the threshold effects, SHAP scatter plots were generated.

**Fig. 3:**
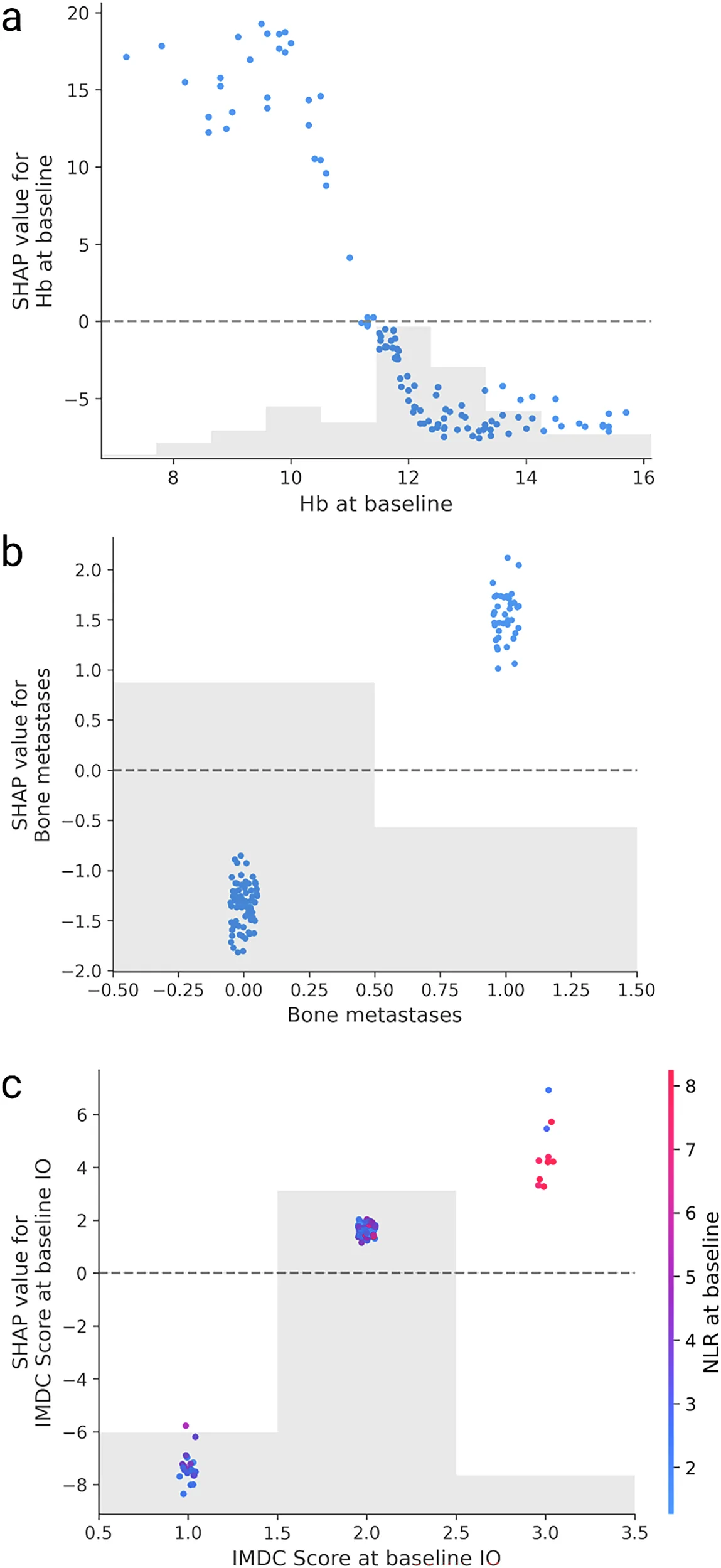
Dependence plots illustrating feature effects on OS prediction. Scatter plots of RSF-univariate for OS on the test set: **a** hemoglobin; **b** bone metastases; **c** IMDC score at baseline, showing the interaction with NLR in determining the prediction. The x-axis shows the feature value and the y-axis shows the corresponding SHAP value. In (**c**), color indicates NLR values.

The most notable scatter plot displays the non-linear relationship between hemoglobin levels and OS prediction: it reveals a hemoglobin threshold of 11 g/dL, with values above this line associated with notably improved OS predictions (Fig. 3a). Similar dependence plots confirm these patterns for other relevant features, such as bone metastases (Fig. 3b).

Moreover, scatter plots can be colored with respect to another feature, to inspect their relationship and to explore how their interaction affects the prediction. Figure 3c shows a scatter plot from the RSF model, depicting how the IMDC score at baseline and NLR influence predictions of OS: it confirms that higher IMDC scores at baseline and elevated NLR values are associated with increased SHAP values, indicating an increased mortality risk.

This visualization supports the model’s capacity to capture complex and clinically relevant relationships between baseline variables and survival outcomes, in particular the effects of systemic inflammation and prognostic scoring on long-term survival in mRCC patients treated with immunotherapy.

### Comparison with single biomarker - Hb

**Fig. 4:**
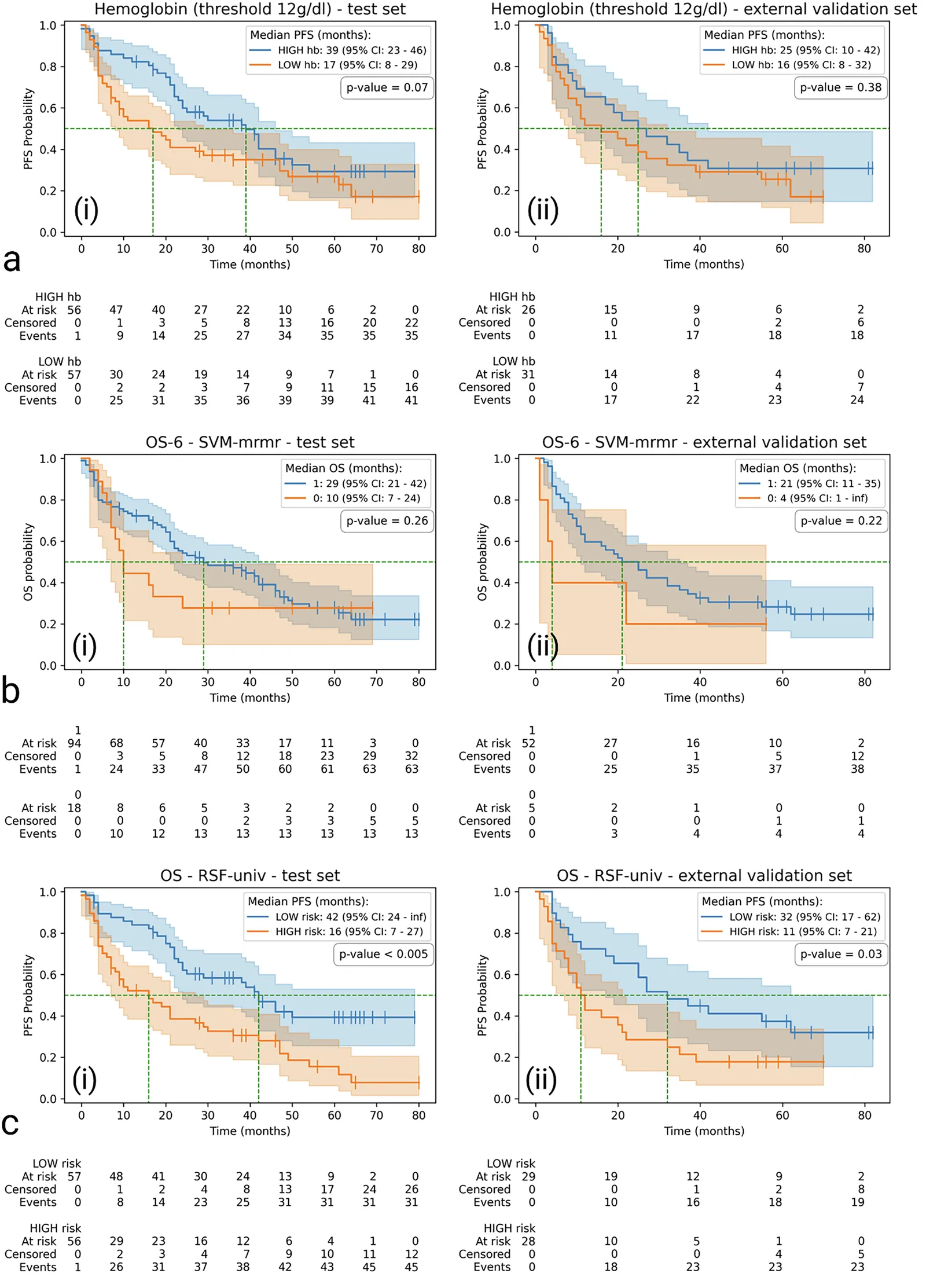
Comparison of patient risk stratification approaches. Patient stratification using (**a**) hemoglobin as a single biomarker in the test (i) and external validation (ii) cohorts; (**b**) predicted classes from the SVM-MRMR model for OS-6 in the test (i) and external validation (ii) cohorts; and (**c**) low- versus high-risk groups identified by the median risk score from the univariate survival RSF model in the test (i) and external validation (ii) cohorts. *P*-values were calculated usingthe log-rank test.

A comparison of the performance of a single biomarker (hemoglobin, feature selected as one of the most important for the survival model) was performed against the best survival model in stratifying patients into high- and low-risk groups in both the test and external validation cohorts (Fig. 4). Patients were stratified based on: hemoglobin value below or above 12 g/dL; the binary classification label (0/1) assigned by the model for the OS-6 outcome; and the continuous risk score assigned by the survival model, dichotomized at the median risk of the population.

Hemoglobin alone was not able to significantly stratify the population into two groups in either cohort. Similarly, the classification model SVM-mrmr for OS-6 did not achieve statistically significant stratification in either the test or the external validation cohort. The RSF-univ survival model for OS was able to significantly stratify patients based on the predicted risk score, achieving a statistically significant separation in the test cohort (*p*-value < 0.005). In the external validation cohort, its model retained prognostic stratification ability, albeit with a less significant *p*-value of 0.03.

### Survival analysis summary

Across both OS and PFS survival tasks, the top-ranked features contributing to model predictions were NLR, PLR, SII, IMDC score at diagnosis and baseline, hemoglobin, lymphocytes, bone metastases and KPS (Table 4).

**Table 4:** Most important features for survival analysis models

Model	Endpoint	c-index test set	Most relevant features									
RSF-univariate	OS	0.68	NLR	PLR	SII	Hb	Lym	Baseline IMDC	Diagnosis IMDC	KPS	Bone mts	-
EST	OS	0.68	NLR	PLR	SII	Hb	Lym	Baseline IMDC	-	KPS	Bone mts	Neph
SSVM-univariate	PFS	0.66	-	PLR	SII	-	Lym	Baseline IMDC	Diagnosis IMDC	KPS	Bone mts	-
EST	PFS	0.65	NLR	PLR	-	Hb	Lym	Baseline IMDC	Diagnosis IMDC	KPS	Bone mts	Neph

*RSF* random survival forest, *SSVM* survival support vector machine, *EST* extra survival trees, *PFS* progression-free survival, *OS* overall survival, *NLR* neutrophils-to-lymphocytes ratio, *PLR* platelets-to-lymphocytes ratio, *SII* systemic immune-inflammation index, *Hb* hemoglobin, *Lym* lymphocytes, *IMDC* International Metastatic RCC Database Consortium, *KPS* Karnofsky performance status, *mts* metastases, *Neph* nephrectomy.

## Discussion

Understanding and predicting clinical benefit to immunotherapy in mRCC remains a critical challenge, given the heterogeneity of clinical outcomes and the lack of universally accepted predictive biomarkers. The application of AI methods in oncology is increasingly being explored to identify prognostic factors and optimize treatment strategies, particularly in the field of immunotherapy, where traditional biomarkers often fall short. These AI models have the potential to surpass traditional prognostic tools by capturing complex and multidimensional data, thereby facilitating personalized treatment strategies. Despite these promising developments, challenges remain in integrating AI models into clinical practice, including issues related to data heterogeneity, model interpretability and the need for external validation.

This study presents an explainable ML-based framework to predict clinical outcomes in mRCC patients treated with ≥ 2^nd^ line nivolumab by using a large real-world cohort from the Meet-URO 15 study. The original Meet-URO 15 study proposed a clinically applicable prognostic score based on IMDC score, NLR and the presence of bone metastases – the Meet-URO score^6^. In this ML-driven analysis, the NLR and IMDC risk group confirmed their prognostic relevance, while the presence of bone metastases maintained a role of prognostic interest. As such, the present analysis supports the reliability of the Meet-URO score^6^, a model previously developed in the same population, by demonstrating that, even in a more complex approach compared to traditional statistical approaches, its core prognostic indicators remain robust.

Moreover, this ML-based analysis enabled the inclusion and assessment of additional inflammatory variables such as SII and PLR, which showed strong prognostic significance, consistent with the previous analysis in the same population^6^. From a biological perspective, the prognostic relevance of these composite inflammatory indices is supported by several immunologic mechanisms: elevated neutrophil counts are associated with tumor-promoting inflammation and immunosuppression, as neutrophils can release mediators such as arginase-1 and reactive oxygen species that inhibit T-cell activation and facilitate tumor immune evasion^21,22^; platelet imbalance may further contribute to tumor progression by promoting angiogenesis and protecting circulating tumor cells from immune surveillance. Conversely, the lymphopenia often reported in these patients reflects an impaired adaptive immunity—which is particularly relevant in the context of immune checkpoint inhibitors, whose therapeutic activity relies on the presence of functional anti-tumor T-cell responses^23^.

These results are not only consistent with, but in some respects exceed, those reported in previous studies applying AI techniques to mRCC treated with immunotherapy^17–20^.

Barkan et al. analyzed 322 patients treated with different first-line therapies, including 24 patients receiving immune-based combinations (7.4%), achieving good predictive performance with AUCs of 0.79 and 0.77 for 3- and 5-year OS prediction, using ensemble ML methods considering clinical and treatment features^18^. Despite our analysis focused on a more clinically heterogeneous cohort of pre-treated patients, where prediction is inherently more challenging, our models showed good performance (AUC 0.68 for OS-24 on the test set). In addition, our study was conducted in a larger (*N* = 571) and therapeutically homogeneous population (only nivolumab), also including the aforementioned inflammatory markers, making our model more applicable in mRCC patients receiving immunotherapy. Another important difference is that our approach applied explainability methods (SHAP), providing interpretability of model outputs.

Gupta et al. developed a Bayesian network model based on CheckMate 025 data (*N* = 803), considering not only baseline clinical data (AUC = 0.74 for OS-12) but also intermediate clinical outcomes (response or progression at 12 months) to enhance prognostic performance (AUC = 0.86 for OS-12)^17^. In 2023, Xiao et al. published an abstract on the development of a deep learning model based on transcriptomic data from 3 cohorts (about 850 patients), achieving very high performance for predicting PFS at 6 months (AUC 0.89 in the test set), which improved with the combination of immune cells infiltration biomarkers (AUC 0.94). Their reliance on complex genomic profiling translated into a biological advantage in the patients’ characterization, but also prevented immediate clinical translation; moreover, it is well known how PFS is not the best survival outcome to consider for immune-based therapies in mRCC^24^.

In contrast to these studies, our models predict survival and disease control using only clinical variables and scores derived from them, offering similar prediction (AUC = 0.68 versus 0.71 for OS-24) and applicability before treatment initiation, and without longitudinal data dependency. Furthermore, incorporating composite indices (e.g., NLR) rather than the simple immune cell count (e.g., neutrophils) and the use of SHAP provided additional biological insight and clinical transparency.

The Meet-URO score itself was not included as a candidate feature in the machine learning models, being a composite score derived from variables - IMDC score, NLR, bone metastases—that were already analyzed individually; including both the composite score and its individual components could introduce redundancy and collinearity within the feature set. Nevertheless, the consistent identification of these variables among the most important predictors in our models indirectly supports the prognostic relevance of the Meet-URO score.

Elgebaly et al. analyzed the largest cohort of mRCC patients receiving first-line immunotherapy (*N* = 4895) using gradient boosting algorithms, identifying clinical features, including tumor grade, prior surgery and age as major predictors^20^. Interestingly, in our analysis age did not emerge among the most relevant features for OS. This finding should be interpreted cautiously, as patients receiving nivolumab in routine practice may represent a clinically selected population in whom chronological age alone is less informative than factors related to biological fitness, inflammatory status, and disease burden; in particular, frailer and more comorbid elderly patients may have been less likely to receive immunotherapy.

Several limitations of our study should be acknowledged. First, the retrospective real-world nature of the Meet-URO 15 dataset may have introduced potential sources of bias, including missing information, selection bias, and heterogeneity in the timing of radiological assessment and clinical management. Moreover, because the study population was derived from 34 Italian oncology centers, inter-center variability may also have contributed to dataset heterogeneity. Although the dataset was centrally curated and harmonized before analysis, such procedures cannot fully exclude center-specific bias or batch effects. Second, the present work was designed as a prognostic model development and validation study on a pooled multicenter cohort, rather than as a comparative analysis across participating institutions. As a result, detailed center-level analyses were beyond its scope, limiting the possibility of formally quantifying site-related heterogeneity. Also, while imputation and cross-validation strategies were used to mitigate the effects of missing data and overfitting, prospective validation remains necessary.

Importantly, external validation was performed on an independent single-center cohort within the same national setting, and included a relatively limited number of patients; as such, the lower performance observed in this cohort may partly reflect institutional differences in unmeasured center-specific factors. Although this approach allowed us to test the models in an independent dataset, larger multicenter external validations will be necessary to better assess model transportability and generalizability before routine clinical use.

Nevertheless, this study also presents several strengths. The use of a large-scale and real-world cohort ensures a representative sample reflective of current clinical practice. The consistency between the variables identified by our models and those by the Meet-URO 15 study^6^, reinforces the robustness and biological validity of both approaches. The application of multiple ML algorithms with rigorous cross-validation and external testing further supports the reliability of our results.

SHAP-based explainability and the reliance on easily accessible clinical and laboratory data enhance the practical utility of the models, facilitating their integration into real-world clinical decision-making.

Future perspectives include mainly the prospective validation of these models (Meet-URO score and ML model) in a large-scale retrospective and/or prospective analysis, as well as the integration of additional data types such as radiomics or genomics. Expanding the model to other therapeutic scenarios, including combination treatments or first-line settings, could further enhance its utility.

A large-scale ambispective study on 1^st^ line systemic strategies (ICI-ICI and ICI-TKI combinations and TKI monotherapy) is currently ongoing, reaching more than 1800 patients at the datalock of September 2025^25^. In the context of this study, we will perform extensive external validation of the AI-based model developed in the present paper.

In conclusion, this ML-based analysis explores an interpretable and clinically relevant framework for predicting immunotherapy outcomes in patients with mRCC. Our findings are consistent with the prognostic role of variables previously identified in the Meet-URO 15 study, including the Meet-URO score^6^, and provide additional insight into individualized risk stratification through explainable models. Moreover, the results suggest that integrating multiple clinical and immunological features may outperform single-variable approaches in predicting response to immunotherapy, in agreement with emerging evidence in the field.

From a clinical perspective, these models may represent a step toward the implementation of machine learning tools in routine practice, particularly as clinical decision support systems integrated into electronic medical records. By providing individualized and readily interpretable risk estimates before treatment initiation, such approaches could assist clinicians in refining therapeutic choices for patients with metastatic renal cell carcinoma. While the proposed models illustrate the potential of machine learning approaches, further validation and prospective studies are required before any integration into routine practice. Overall, our study contributes to the growing effort to translate artificial intelligence into clinically actionable strategies - ultimately aimed at improving precision oncology.

## Methods

**Fig. 5:**
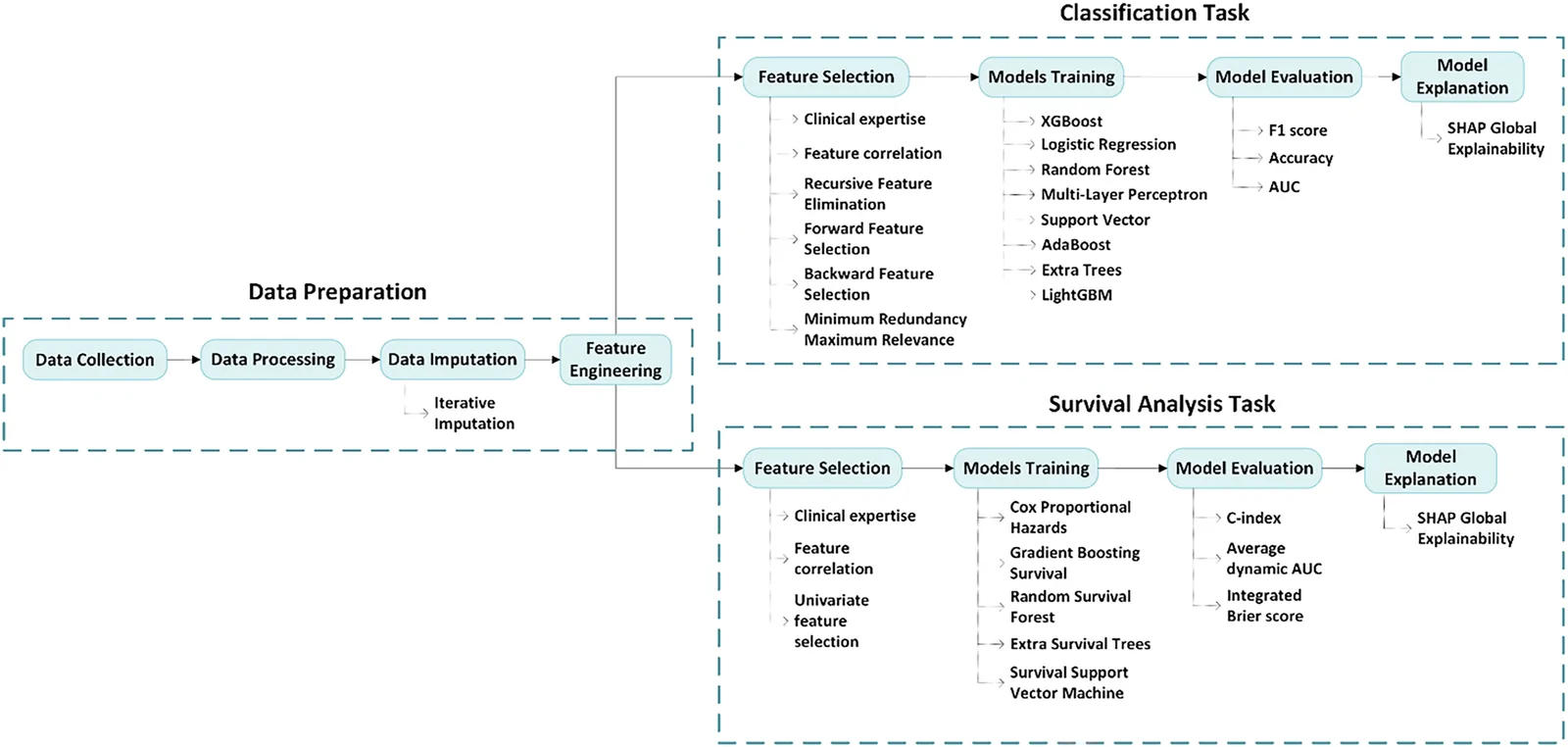
Methodology workflow. Overview of the analytical pipeline, including data collection, dataset curation, data processing and imputation, feature selection, model training, internal and external evaluation, and model explanation.

The multi-step analysis included data collection, processing, imputation and subsequent feature selection (Data preparation); then, model training, internal and external evaluation and model’s explanation (Model development) (Fig. 5).

### Data collection (Study population)

The Meet-URO 15 is a multicentric retrospective study, which enrolled mRCC patients receiving ≥ 2^nd^ line nivolumab from May 2016 to January 2019 across 34 Italian centers. Details of the study design were reported in the primary publication^6^. The dataset contains real-world data (RWD) including patient and tumor characteristics, laboratory exams and treatment information. Patients from the Istituto Oncologico Veneto (IOV IRCCS, Padova) were used as an independent external validation cohort to evaluate the generalizability of the models in a dataset originating from a separate institution. The study was approved by the Regional Ethical Committee of Liguria (registration number 068/2019) and was conducted across 34 Italian centers. The study was performed in accordance with the Declaration of Helsinki, Good Clinical Practice guidelines and local ethical regulations. Written informed consent was obtained from all participants where required by local regulations.

### Dataset curation

According to clinical expertise, an initial hypothesis-driven feature selection was performed, analyzing clinical, disease and treatment characteristics at the time of RCC diagnosis, at mRCC diagnosis and at baseline before nivolumab initiation. Finally, the features were encoded by converting textual data to numerical or categorical data.

### End point

The study aim was to apply ML techniques, both classification and survival analysis tasks, to predict the effectiveness of ≥2^nd^ line immunotherapy in mRCC patients in terms of short-term and long-term outcome prediction and to identify the most relevant prognostic factors influencing the treatment benefit.

For the ML classification analysis, the binary outcomes considered were disease control rate (DCR), overall survival (OS) at 6, 18, and 24 months, and progression-free survival (PFS) at 3 and 9 months, outcomes defined according to the Response Evaluation Criteria in Solid Tumors (RECIST)^26^.

Particularly, for the prediction of DCR, the categorization of patients into responders (R) (Complete Response, Partial Response, or Stable Disease) versus non-responders (NR) (Progressive Disease) as best response regardless of timing of response, was performed. For the other outcomes, the months-threshold was considered for the progression and vital status assessment. Specifically, patients were labeled as events if the outcome occurred strictly before the threshold, and as event-free if they were still alive or progression-free at the threshold. Patients censored before the fixed time threshold were excluded from the corresponding analysis, as their status at that timepoint could not be determined.

For the ML survival analysis, OS and PFS were considered as continuous outcomes. Survival analysis aims to predict time-to-event data and accommodate censored observations, where patients may not have experienced disease progression or death at the end of follow-up, capturing the true nature of longitudinal oncological outcomes.

### Data processing and imputation

All the analysis was conducted in Python v3.11. The data was split into training, testing and external validation sets at ratios of 70%-20%-10%, respectively. The external validation set included only the patients from the Istituto Oncologico Veneto - IOV IRCCS, Padova, Italy, as it represented a single-center dataset kept completely separate from model training and internal testing—thus allowing an institutionally independent assessment of model transportability. The splitting between train and test sets was performed using stratification to maintain the proportion between censored and not censored for OS in survival analysis.

All data preparation steps were performed exclusively on the training set, and the resulting transformations were subsequently applied in the same way to the test and external validation sets, ensuring no information leakage from held-out data.

Regarding missing data, analysis of the missingness patterns revealed that missing values were predominantly dependent on the center of data collection rather than on the missing values themselves, supporting the Missing At Random (MAR) assumption conditionally on the center. Features having a high amount of missing values were excluded from the analysis. The remaining missing values were imputed using the Iterative Imputer^27^, which estimates missing values by iteratively modeling each feature as a function of all others, including the feature indicating the center of data collection, thus explicitly accounting for the center-dependent missingness mechanism.

Following imputation, three inflammatory composite features were computed and added to the feature set: the neutrophil-to-lymphocyte ratio (NLR), the platelet-to-lymphocyte ratio (PLR), and the systemic immune-inflammation index (SII). Categorical features were examined to ensure a sufficient number of instances was present in each category. For numerical continuous features, logarithmic transformation was applied to skewed features to approximate normality, followed by standardization of all numerical features through mean removal and scaling to unit variance.

### Feature selection

Pearson correlation was used to assess inter-feature correlation, with a threshold of 80%; features exceeding this threshold were removed after consultation with clinicians.

For classification, two kinds of feature selection methods were used, one Filter method, Minimum Redundancy Maximum Relevance (MRMR)^28^, and three Wrapper methods: Forward Feature Selection (FFS), Backward Feature Selection (BFS), and Recursive Feature Elimination (RFE)^29^. Wrapper methods are model-related and subsets of features are selected based on their impact on the performance of a specific predictive model, achieving high performance.

Filter methods are not model-related and select features based on their statistical properties, independently of any machine learning model, using predefined criteria.

RFE was not applied to Logistic Regression, Multilayer Perceptron, Support Vector Machine and Adaboost, as these models either do not natively expose feature importance scores or coefficients in a form suitable for the iterative feature elimination procedure, or their feature ranking is insufficiently stable across elimination steps to guarantee a reliable selection.

For survival analysis, univariate feature selection was applied as a model-specific technique. This approach evaluates each feature independently by training the model and computing a chosen performance metric, concordance index (c-index). Features with c-index exceeding a predefined threshold (0.55) were retained for final model development, while others were excluded. This threshold was chosen to retain variables with at least minimal discriminative ability beyond chance, while avoiding overly restrictive filtering. This method allows maintaining the features that have predictive capability by themselves and selecting the best features for the specific model.

### Model training and evaluation

For classification analysis, eight ML models were employed: XGBoost (XGB)^30^, Logistic Regression (LR)^31^, Random Forest (RF)^32^, Multilayer Perceptron (MLP)^33^, Support Vector Machine (SVM)^34^, Adaboost (AB)^35^, Extra Trees (ET)^36^, LightGBM (LGBM)^37^.

Feature selection and hyperparameter tuning were performed jointly within a unified pipeline, combining feature selection with the model of interest. This pipeline was provided as input to RandomizedSearchCV using 5-fold cross-validation stratified with respect to the outcome, with the weighted F1-score as optimization target. Each model was also trained and evaluated without the feature selection step, to assess the impact of feature selection on model performance. The primary evaluation metric was the weighted F1-score; balanced accuracy (ACC) and Area Under the Receiver Operating Characteristic Curve (AUC) were additionally computed as secondary metrics.

For survival analysis, six ML models were tested: baseline model Cox Proportional Hazards (CPH), Survival Trees (ST)^38^, Random Survival Forest (RSF)^39^, Gradient Boosting (GB)^40^, Extra Survival Trees (EST), Survival Support Vector Machine (SSVM)^41^.

Similarly, feature selection and hyperparameter tuning were performed jointly within a unified pipeline provided as input to RandomizedSearchCV using 5-fold cross-validation stratified based on the event indicator (progression or death versus censored, for PFS and OS, respectively), with the C-index as optimization target. Each model was also trained and evaluated without the feature selection step. The primary evaluation metric was the C-index; time-dependent AUC and Integrated Brier Score (IBS) were computed as secondary metrics^42^.

The best combination of feature selector and ML model, for both classification and survival analysis tasks, was then tested on the external validation set.

### Model explanation

After the identification of the best combination of feature selector and model for each ML task, Shapley additive explanations (SHAP) approach^43^ was applied to enhance explainability and provide insights into the prediction process. This approach allows evaluating what is the contribution of each feature to the considered outcome.

SHAP, a state-of-the-art explainability method, assigns each feature a “Shapley value,” representing its contribution to the model’s predictions. SHAP values were calculated for both the test and external validation sets^44^.

In particular, we focused the analyses on the global explainability approach to understand the overall behavior of the model and identify the most important features driving its prediction capability. Beeswarm, scatter and decision plots were used to identify overall trends, feature importance, and relationships among features across the dataset. These visualizations aimed to provide a comprehensive understanding of the model’s behavior and the relative influence of features on predictions. This method assigns each feature a value representing its impact on the model’s output.

The SHAP beeswarm plot provides a global explanation of the model by highlighting the most important features and their impact on predictions. Features are ranked on the vertical axis according to their importance, defined as the mean absolute SHAP value across all instances. Each point represents an individual patient, positioned along the horizontal axis based on the corresponding SHAP value, which quantifies the contribution of that feature to the prediction. In classification tasks, negative SHAP values indicate an association with the negative class (non-responder), while positive values indicate an association with the positive class (responder). In survival analysis, negative SHAP values correspond to lower risk (better outcome), whereas positive values indicate higher risk (worse outcome). Feature values are encoded by color, with blue representing low values and pink representing high values.

The SHAP scatter plot provides a global explanation of the effect of a single feature on model predictions. In this plot, feature values are shown on the horizontal axis, while the corresponding SHAP values are reported on the vertical axis, representing the contribution of that feature to the prediction. Each point corresponds to an individual instance. To capture feature interactions, points can be colored according to the values of a secondary feature, with blue indicating low values and pink indicating high values.

The SHAP decision plot illustrates how individual feature contributions combine to form each prediction. The horizontal axis represents the model output, while features are displayed on the vertical axis, typically ordered by decreasing importance. Each line corresponds to a single instance and is initialized at the model’s expected (base) value. Moving upward along the plot, SHAP values for each feature are cumulatively added, leading to the final predicted value.

In the standard implementation, each line is colored according to the predicted outcome (e.g., blue for lower risk and pink for higher risk). In this study, we adapted the visualization so that the color of each segment reflects the value of the corresponding feature, ranging from green (low values) to yellow (high values), thereby providing additional insight into feature-specific contributions along the prediction path.

## Supplementary information

Supplementary files

## Data Availability

The datasets analyzed during the current study are not publicly available due to privacy and ethical restrictions but are available from the corresponding author upon reasonable request.

## Code availability

The underlying code used for the analyses in this study is available on this link: https://github.com/AI-ON-Laboratory/Meet-URO_15_AI.
