# Supplementary material for: Explainable machine learning to predict immunotherapy outcomes in metastatic renal cell carcinoma - Meet-URO 15-AI study
Source: NPJ Precis Oncol. 2026 Jul 2;10:402. doi: 10.1038/s41698-026-01576-x (PMC13691107; doi:10.1038/s41698-026-01576-x)
Supplement: Supplementary file 1 [file 41698_2026_1576_MOESM1_ESM.pdf]

Supplementary Results

	Outcome		Overall dataset		Training set		Test set		External Validation set	
**Classification**	DCR	non-responder	568	217	398	154	113	41	57	22
		responder		351		244		72		35
	OS-6	< 6	560	101	390	66	113	23	57	12
		≥ 6		459		324		90		45
	OS-18	< 18	542	217	376	150	109	41	57	26
		≥ 18		325		226		68		31
	OS-24	< 24	536	271	370	188	109	53	57	30
		≥ 24		265		182		56		27
	PFS-3	< 3	570	103	399	76	114	18	57	9
		≥ 3		467		323		96		48
	PFS-9	< 9	569	311	398	218	114	60	57	33
		≥ 9		158		180		54		24
**Survival Analysis**	OS	censored	571	187	400	134	114	38	57	15
		dead		384		266		76		42
	PFS	censored	570	69	399	50	114	10	57	9
		progressive disease/dead		501		349		104		48

Supplementary Table 1. Dataset preparation according to the ML tasks, sets and outcomes. The outcomes with the best class balancing are OS-24 and PFS-9.

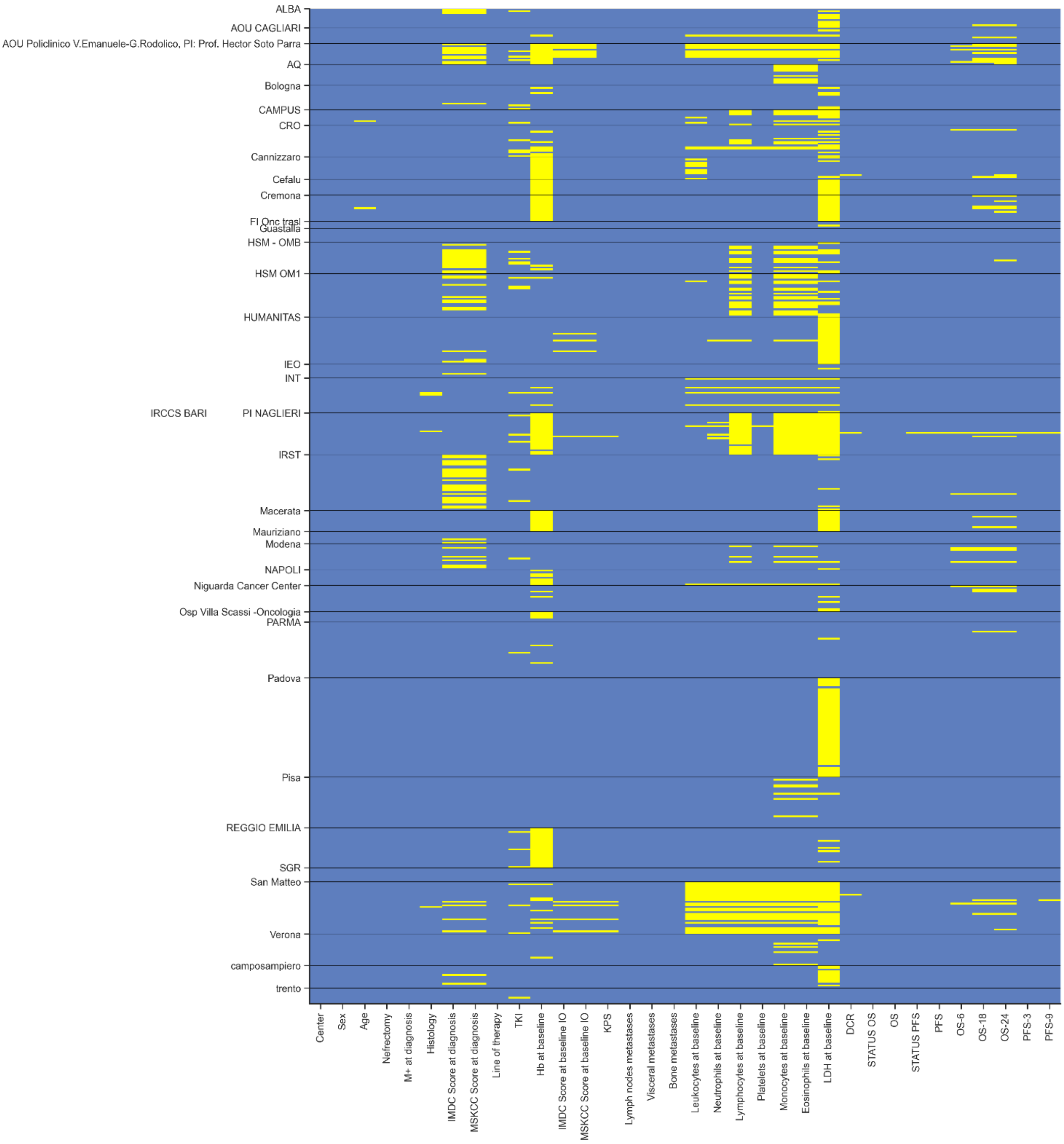

Supplementary Figure 1. Missing values stratified by center. Each row corresponds to a center and each column to a feature. Yellow stripes indicate the presence of missing values for the corresponding feature-center combination, while blue cells indicate complete data.

**Patients' characteristics**	Age
	Sex
	KPS
**Tumor history**	Nephrectomy
	Histology
	Metastatic at diagnosis

	MSKCC score at diagnosis
	IMDC score at diagnosis
**Tumor presentation**	Lymphnodes metastases
	Visceral metastases
	Bone metastases
**Cancer data at baseline**	Previous TKI
	Line of immunotherapy
	MSKCC score at baseline
	IMDC score at baseline
**Laboratory data at baseline**	Hemoglobin
	Leukocytes
	Neutrophils
	Lymphocytes
	Monocytes
	Eosinophils
	Platelets
	LDH
	NLR
	PLR
	SII

Supplementary Table 2. Clinical features identified.

KPS Karnofsky performance status, *MSKCC* Memorial Sloan–Kettering Cancer Center, *IMDC* International Metastatic RCC Database Consortium, *TKI* tyrosine kinase inhibitor, *LDH* lactate dehydrogenase, *NLR* neutrophils-to-lymphocytes ratio, *PLR* platelets-to-lymphocytes ratio, *SII* systemic immune-inflammation index.

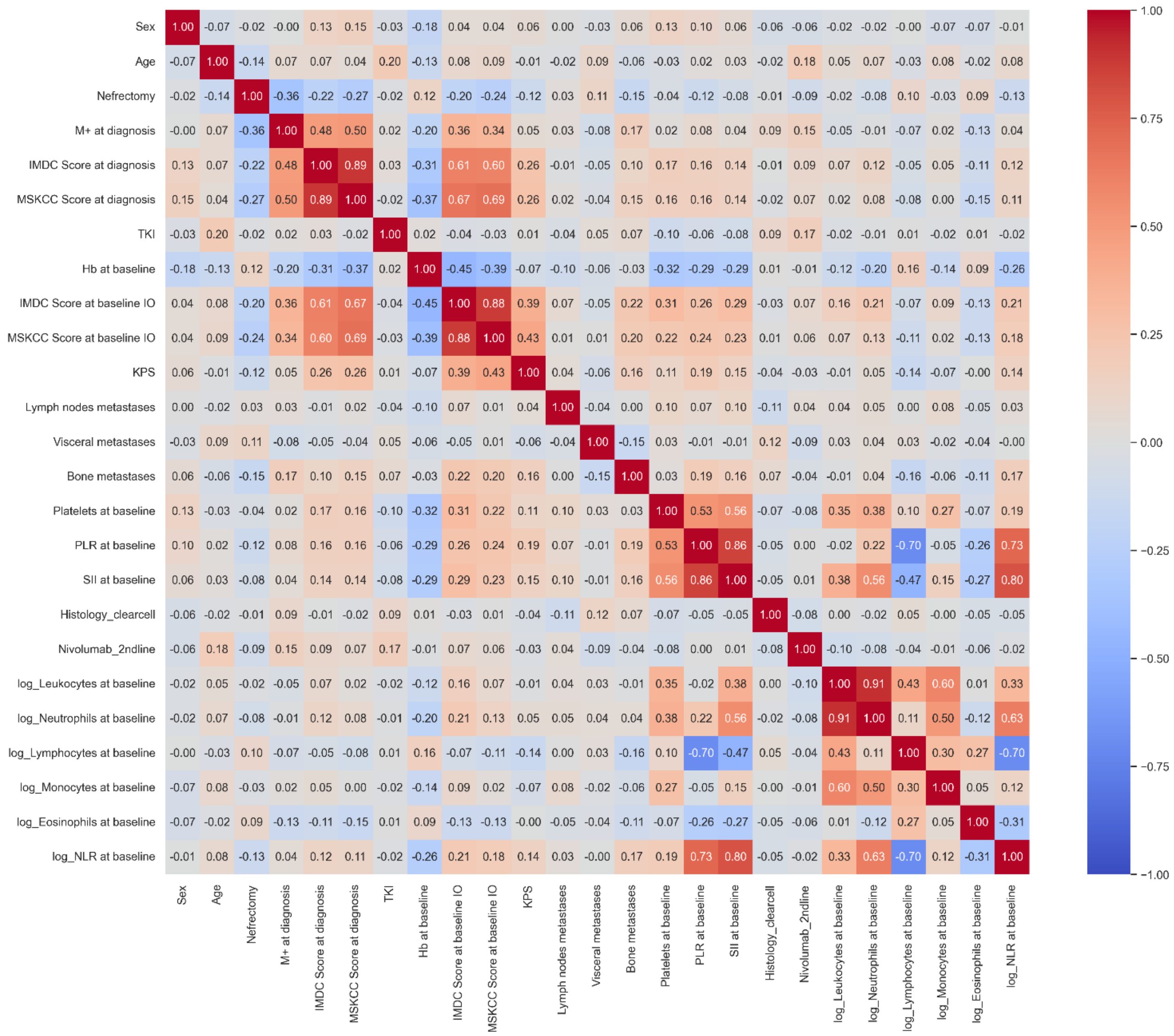

Supplementary Figure 2. Correlation matrix between clinical features identified. MSKCC score is highly correlated with IMDC score, both at diagnosis and at baseline.

Model	Hyperparameters
XGB	max_depth: [2,3]<br>eta: [0.01,0.03,0.3]<br>n_estimators: [30,50,100]<br>reg_lambda': [1,3,8]
LR	C: [1e-4,1e-3,1e-2,0.1,1,10]
RF	max_depth: [2,3]<br>min_samples_leaf: [2,3,4]<br>min_samples_split: [2,3,4]<br>n_estimators: [50,100]
MLP	hidden_layer_sizes: [(10,)]<br>alpha: [0.001,0.01,0.1,1]<br>max_iter: [2000]
SVM	C: [1e-3,0.01,0.1,1]<br>kernel: ['rbf','linear']<br>gamma: [0.01,0.1,1,10,100]

AB	learning_rate: [0.001,0.01,0.1]<br>estimator: [DecisionTreeClassifier(max_depth=i) for i in range(2,4)]<br>n_estimators: [30,50,100]
ET	max_depth: [2,3]<br>min_samples_leaf: [3,4,5]<br>min_samples_split: [2,3,4]<br>n_estimators: [50,100]
LGBM	learning_rate: [0.001,0.01,0.1,1]<br>max_depth: [2,3]<br>num_leaves: [5,10,20,31]<br>n_estimators: [30,50,100]

Supplementary Table 3. List of hyperparameters explored for each classification model.

Disease Control Rate (DCR)

Among the tested models, the best performance for DCR prediction was achieved by the ET model trained with BFS Backward Feature Selection (ET-BFS), utilizing 22 features. On the test set, the model achieved an F1-score of 0.64 (**Supplementary Figure 3**), ACC of 0.61 and an AUC of 0.65. However, the model exhibited limited generalizability, with a notable drop in all metrics (F1 = 0.59, ACC = 0.56, AUC = 0.63) on the external validation set .

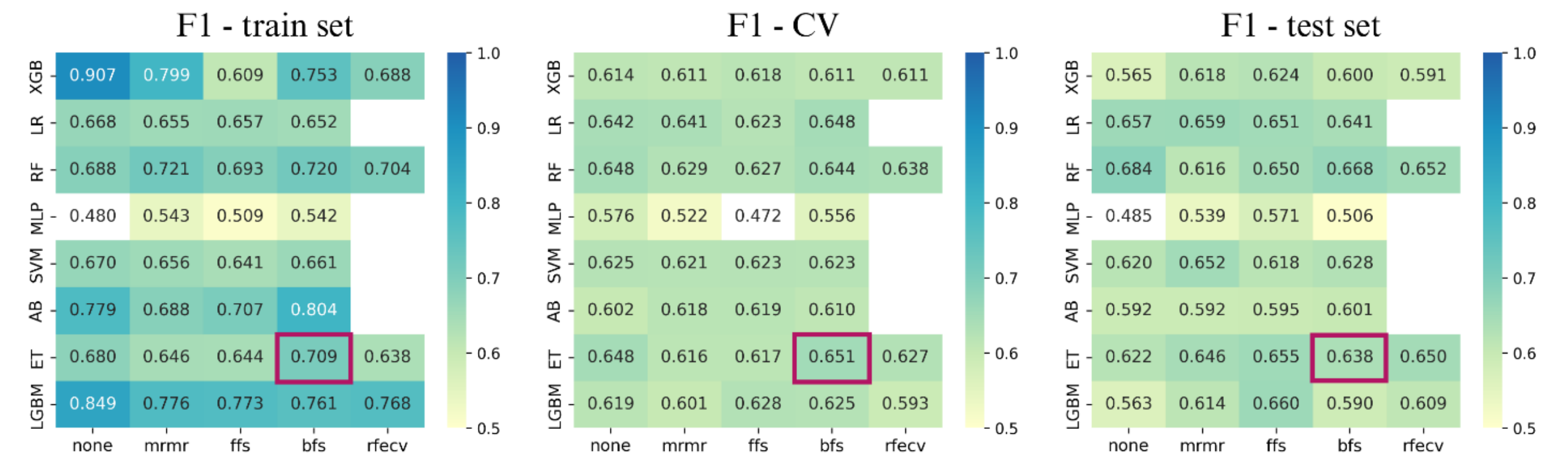

Supplementary Figure 3. Heatmaps of the F1-scores for all the combinations of feature selector and model for DCR.

The combination of model and feature selector was chosen (marked in red) by looking at the CV F1-scores.

Feature importance interpretation via SHAP explainability analysis indicated KPS as the dominating feature. It should be noted that the KPS feature was encoded as 0 for KPS ≥ 80% (good performance status) and 1 for KPS < 80% (poor performance status). Therefore, in the SHAP plot, higher KPS feature values correspond to poorer performance status, and the direction of the association should be interpreted accordingly. Higher SHAP values for KPS were associated with lower encoded values (KPS ≥ 80%), indicating that good performance status contributed to increase the probability of being assigned to the responder class, which is clinically consistent. Presence of bone metastases and being metastatic at diagnosis were associated with a higher predicted probability of worse response, whereas low IMDC score at baseline and previous nefrectomy were linked to the prediction of better disease control outcomes (Supplementary Figure 4a).

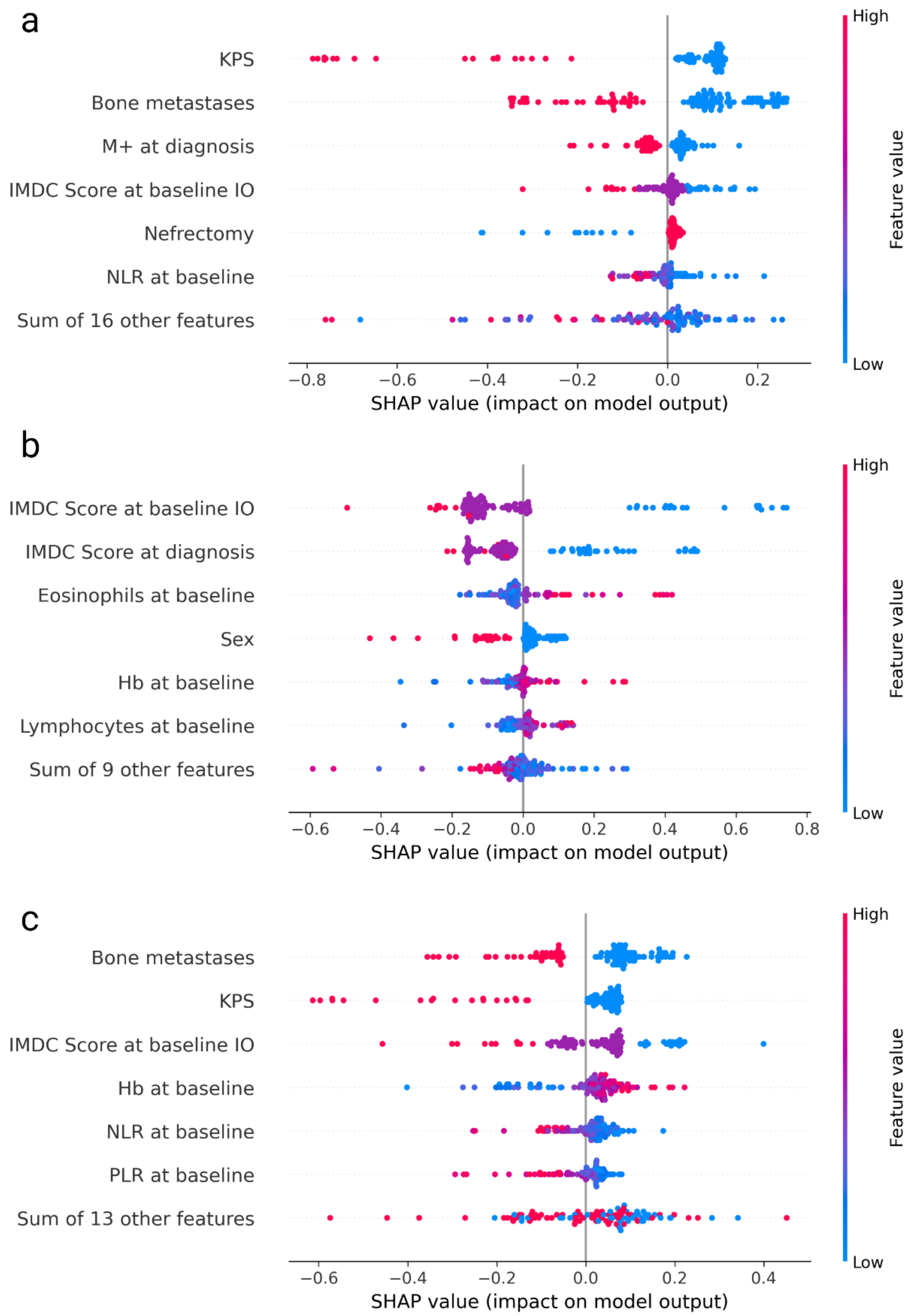

Supplementary Figure 4. SHAP beeswarm plots for the test set population of: (a) ET-BFS for DCR; (b) ET-RFE for PFS-9; (c) ET-BFS for OS-18.

Progression-Free Survival at 9 Months (PFS-9)

For 9-month PFS, the top model was ET with RFE (ET-RFE), using 15 features, which performed modestly (F1 = 0.63, ACC = 0.63, AUC = 0.68) with good generalization in the external validation (F1 = 0.67, ACC = 0.66, AUC = 0.69) (**Supplementary Figure 5**).

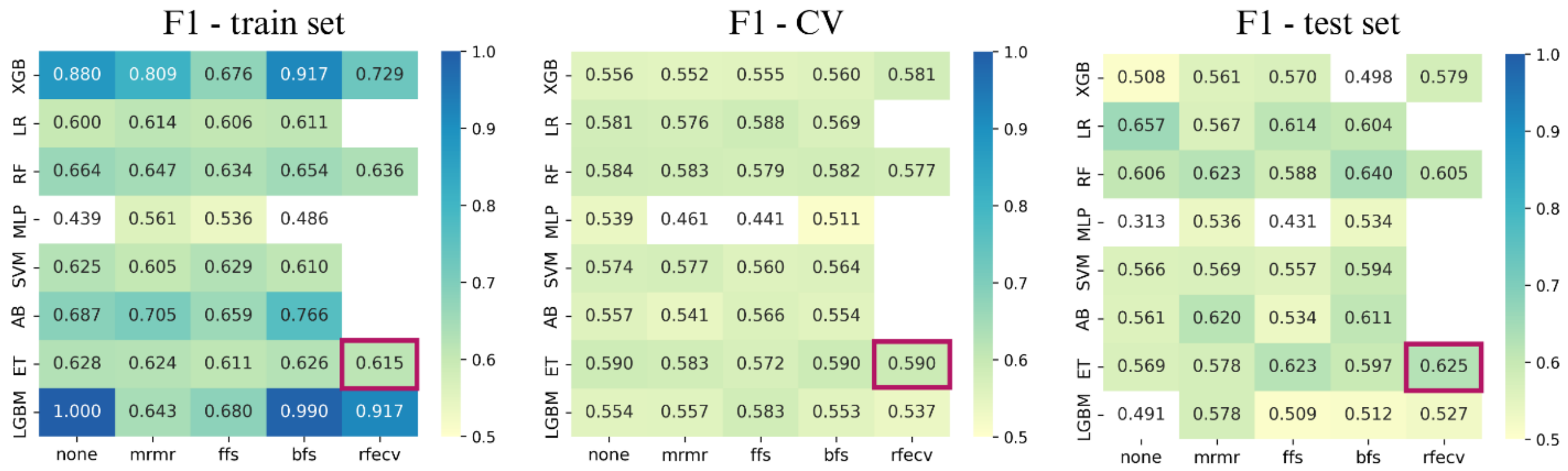

Supplementary Figure 5. Heatmaps of the F1-scores for all the combinations of feature selector and model for PFS-9.

Explainability analysis showed IMDC scores at baseline and diagnosis as the most important features, with low values associated with better outcomes, as well as high values of eosinophils, lymphocytes and hemoglobin. On the contrary, female sex appeared to be linked to the non-responder class (Supplementary Figure 4b).

Overall Survival at 18 Months (OS-18)

18-month OS prediction ET with BFS feature selection (ET-BFS), using 19 features, emerged as the optimal model, producing test set metrics of F1 = 0.74, ACC = 0.72, and AUC = 0.79. External validation showed limited generalization performance (F1 = 0.56, ACC = 0.56, AUC = 0.69) (**Supplementary Figure 6**).

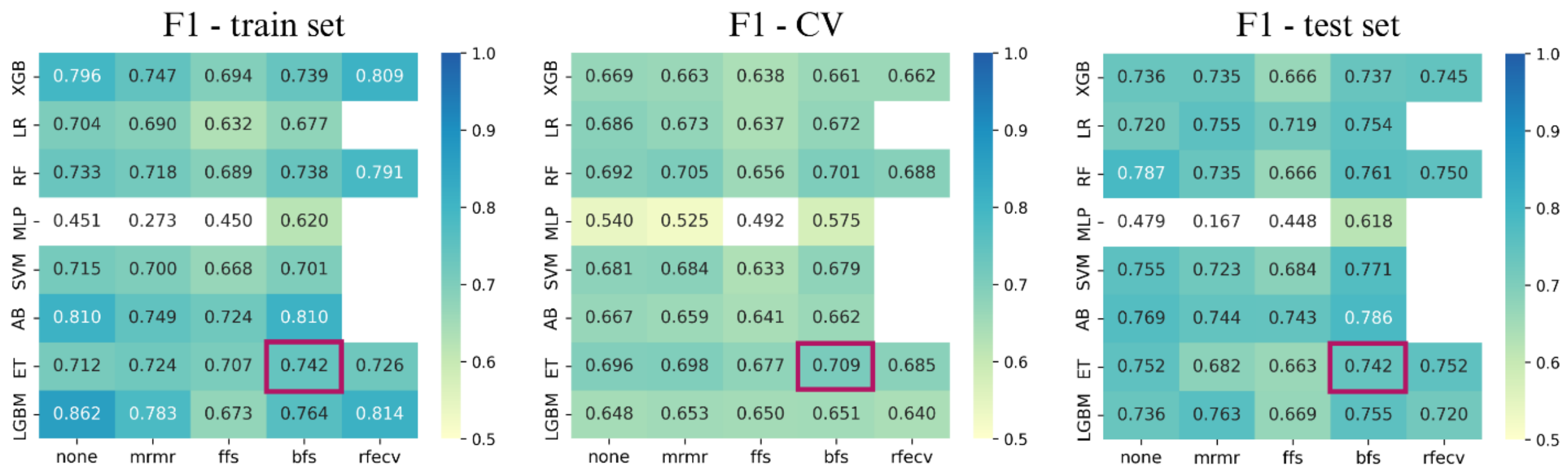

Supplementary Figure 6. Heatmaps of the F1-scores for all the combinations of feature selector and model for OS-18.

SHAP analysis confirmed the relevance of systemic inflammatory indices (NLR and PLR), bone metastases, KPS, Hemoglobin and IMDC score. In particular, higher values of inflammatory indices were associated with a higher predicted risk of mortality at 18 months; on the other hand, high levels of hemoglobin and low baseline IMDC score were associated with positive predictors for survival (**Supplementary Figure 4c**).

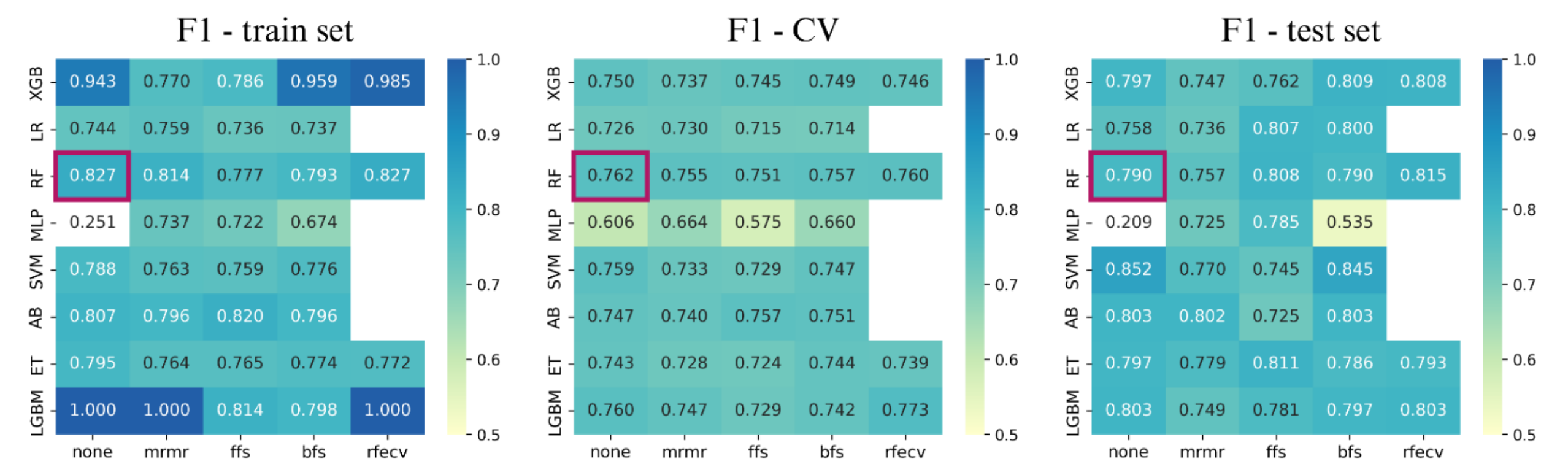

Supplementary Figure 7. Heatmaps of the F1-scores for all the combinations of feature selector and model for PFS-3.

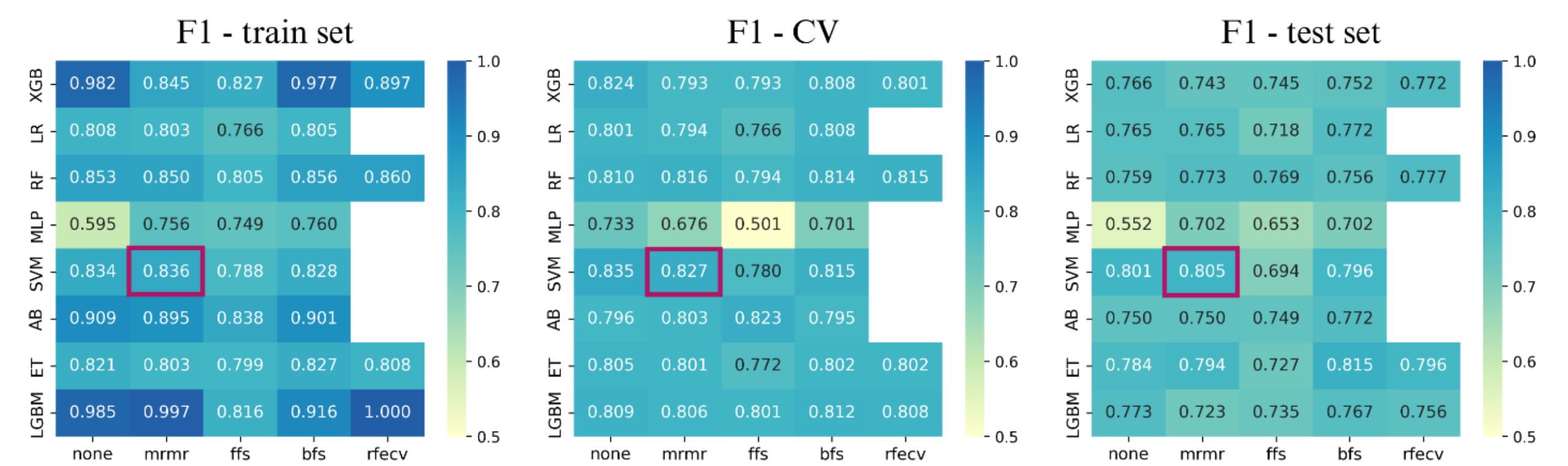

Supplementary Figure 8. Heatmaps of the F1-scores for all the combinations of feature selector and model for OS-6.

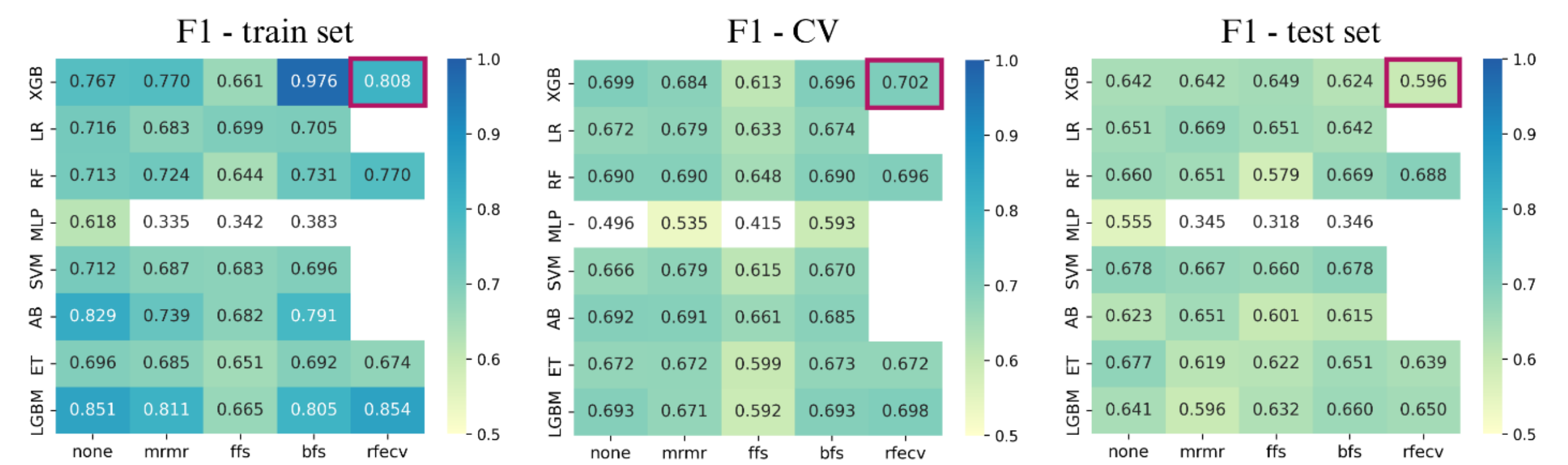

Supplementary Figure 9. Heatmaps of the F1-scores for all the combinations of feature selector and model for OS-24.

DCR

	RFE	FFS	BFS	MRMR
XGB	4	1	21	10
LR	-	4	18	10
RF	19	5	21	10
MLP	-	1	21	10
SVM	-	3	19	10
AB	-	4	22	10
ET	3	3	22	10
LGBM	8	8	20	10

PFS-3

	RFE	FFS	BFS	MRMR
XGB	22	7	21	10
LR	-	2	18	10
RF	20	5	18	10
MLP	-	4	22	10
SVM	-	3	20	10
AB	-	4	22	10
ET	19	4	18	10
LGBM	17	2	21	10

PFS-9

	RFE	FFS	BFS	MRMR
XGB	5	4	19	10
LR	-	3	21	10
RF	11	2	20	10
MLP	-	3	22	10
SVM	-	4	18	10
AB	-	3	22	10
ET	15	4	20	10
LGBM	7	3	22	10

OS-6

	RFE	FFS	BFS	MRMR
XGB	13	5	21	10
LR	-	4	17	10
RF	16	3	22	10
MLP	-	5	21	10
SVM	-	2	20	10
AB	-	4	19	10
ET	19	3	21	10
LGBM	17	2	21	10

OS-18

	RFE	FFS	BFS	MRMR
XGB	7	5	22	10
LR	-	2	19	10
RF	19	3	18	10
MLP	-	2	22	10
SVM	-	5	21	10
AB	-	8	19	10
ET	23	5	19	10
LGBM	20	3	22	10

OS-24

	RFE	FFS	BFS	MRMR
XGB	11	5	22	10
LR	-	4	19	10
RF	22	5	21	10
MLP	-	5	21	10
SVM	-	5	20	10
AB	-	3	18	10
ET	19	5	18	10
LGBM	20	6	22	10

Supplementary Table 4. Number of selected features for all ML models and tasks.

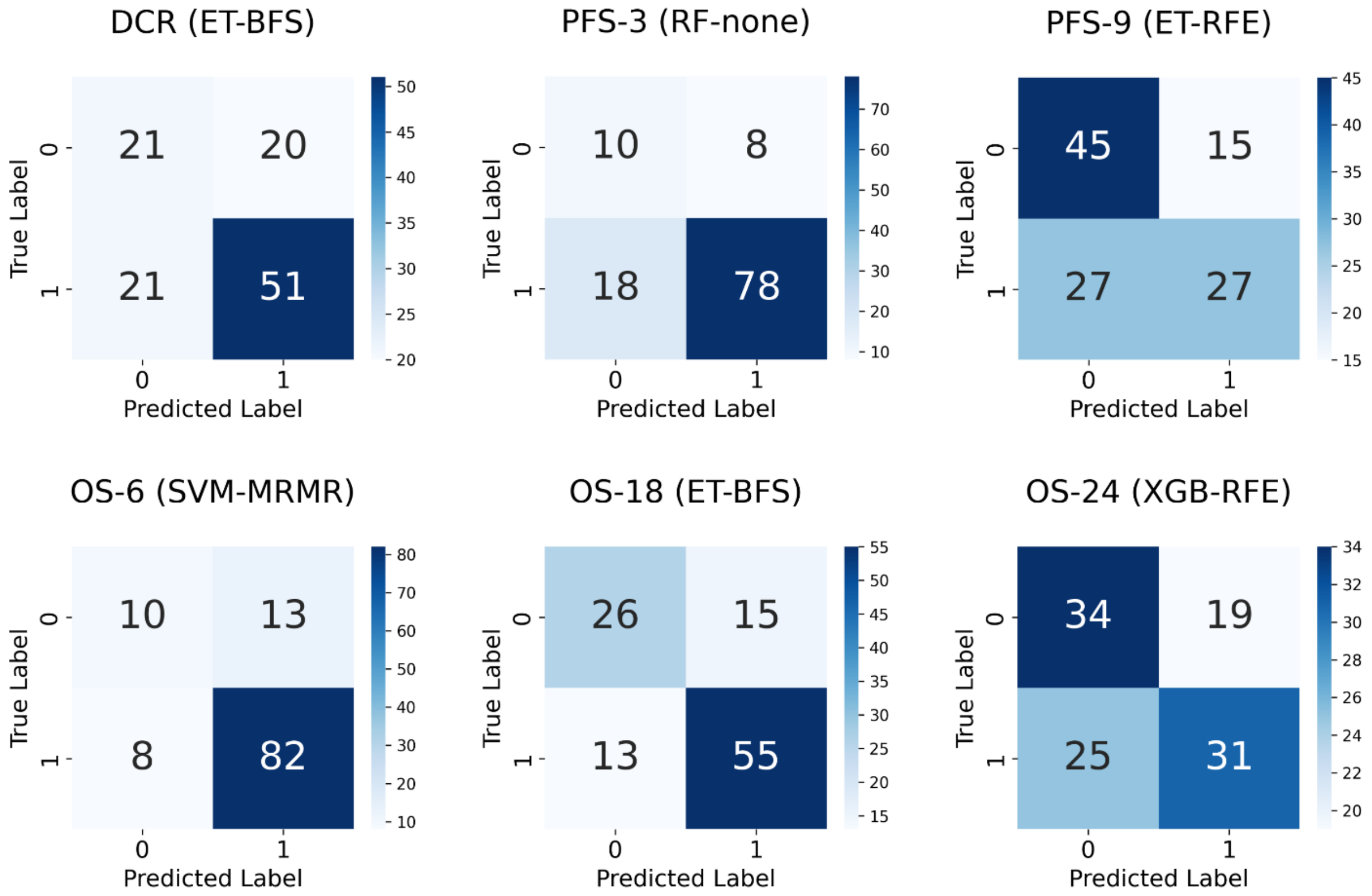

Supplementary Figure 10. Confusion matrices of the best models for each classification task.

Model	Hyperparameters
CPH	n_iter: [5,10,20,50,100,300]<br>alpha: [0.1,0.5,0.7,0.9]<br>ties: ['efron','breslow']
GBS	n_estimators: [200, 300]<br>learning_rate: [0.02, 0.05]<br>max_depth: [2]<br>min_samples_leaf: [20, 30]
RSF	max_depth: [2, 3, 4]<br>min_samples_leaf: [10, 20, 30]<br>min_samples_split: [10, 20, 30]<br>n_estimators: [100, 200]
EST	n_estimators: [200, 400]<br>max_depth: [2, 3, 4]<br>min_samples_split: [10, 20, 30]<br>min_samples_leaf: [10, 20, 30]
SSVM	kernel: ["rbf"]<br>alpha: [1e-3, 5e-3, 1e-2]<br>gamma: [1e-3, 5e-3, 1e-2]<br>tol: [1e-4]<br>max_iter: [2000]

Supplementary Table 5. List of hyperparameters explored for each survival analysis model.

OS - all						PFS – all					
	C-index Train	C-index CV (std)	C-index Test	Mean-AUC Test	IBS Test		C-index Train	C-index CV (std)	C-index Test	Mean-AUC Test	IBS Test
CPH	0.70	0.67 (±0.03)	0.66	0.73	0.19	CPH	0.64	0.58 (±0.06)	0.64	0.69	0.16
GBS	0.74	0.69 (±0.05)	0.68	0.74	0.19	GBS	0.72	0.59 (±0.04)	0.66	0.72	0.16
RSF	0.72	0.69 (±0.05)	0.68	0.75	0.18	RSF	0.69	0.61 (±0.04)	0.65	0.71	0.16
**EST**	**0.70**	**0.69 (±0.06)**	**0.68**	**0.75**	**0.20**	**EST**	**0.65**	**0.62 (±0.05)**	**0.65**	**0.71**	**0.17**
SSVM	0.72	0.69 (±0.04)	0.68	0.74	*	SSVM	0.64	0.60 (±0.06)	0.65	0.71	*
**OS - univariate**						**PFS – univariate**					
	C-index Train	C-index CV (std)	C-index Test	Mean-AUC Test	IBS Test		C-index Train	C-index CV (std)	C-index Test	Mean-AUC Test	IBS Test
CPH	0.69	0.69 (±0.04)	0.68	0.74	0.18	CPH	0.63	0.59 (±0.05)	0.65	0.71	0.16
GBS	0.74	0.68 (±0.04)	0.68	0.74	0.19	GBS	0.68	0.59 (±0.03)	0.63	0.68	0.16
**RSF**	**0.72**	**0.69 (±0.05)**	**0.68**	**0.73**	**0.18**	RSF	0.67	0.58 (±0.03)	0.65	0.70	0.16
EST	0.71	0.69 (±0.06)	0.69	0.76	0.19	EST	0.63	0.59 (±0.04)	0.66	0.72	0.17
SSVM	0.71	0.69 (±0.04)	0.68	0.75	*	**SSVM**	**0.63**	**0.61 (±0.05)**	**0.66**	**0.72**	*

Supplementary Table 6. Modeling results using all features and the reduced univariate-selected feature set. (* For SSVM, Brier score can't be computed.)

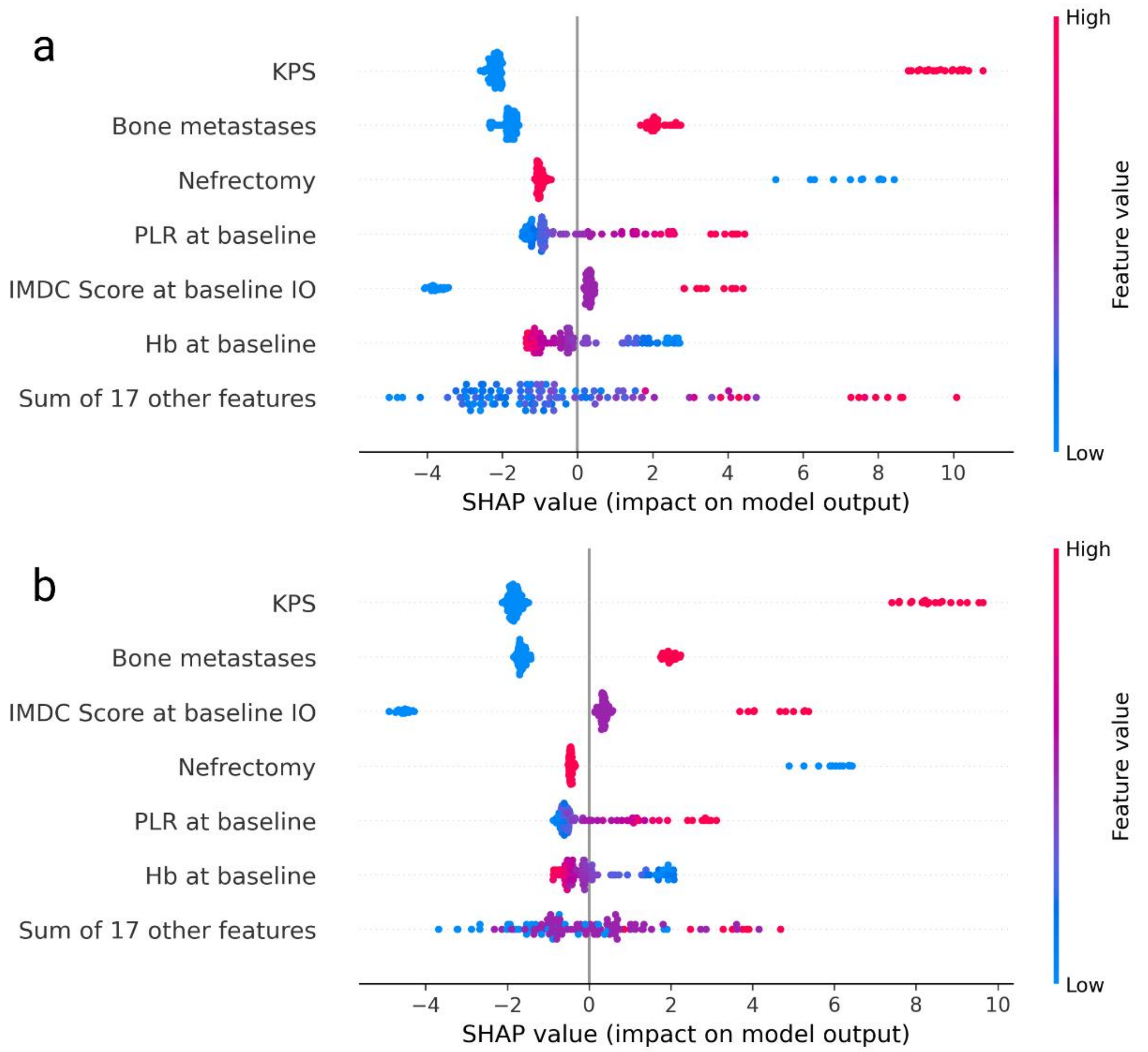

Supplementary Figure 11. Beeswarm plots of survival models applied to the test set: (a) EST-all for OS; (b) EST-all for PFS.

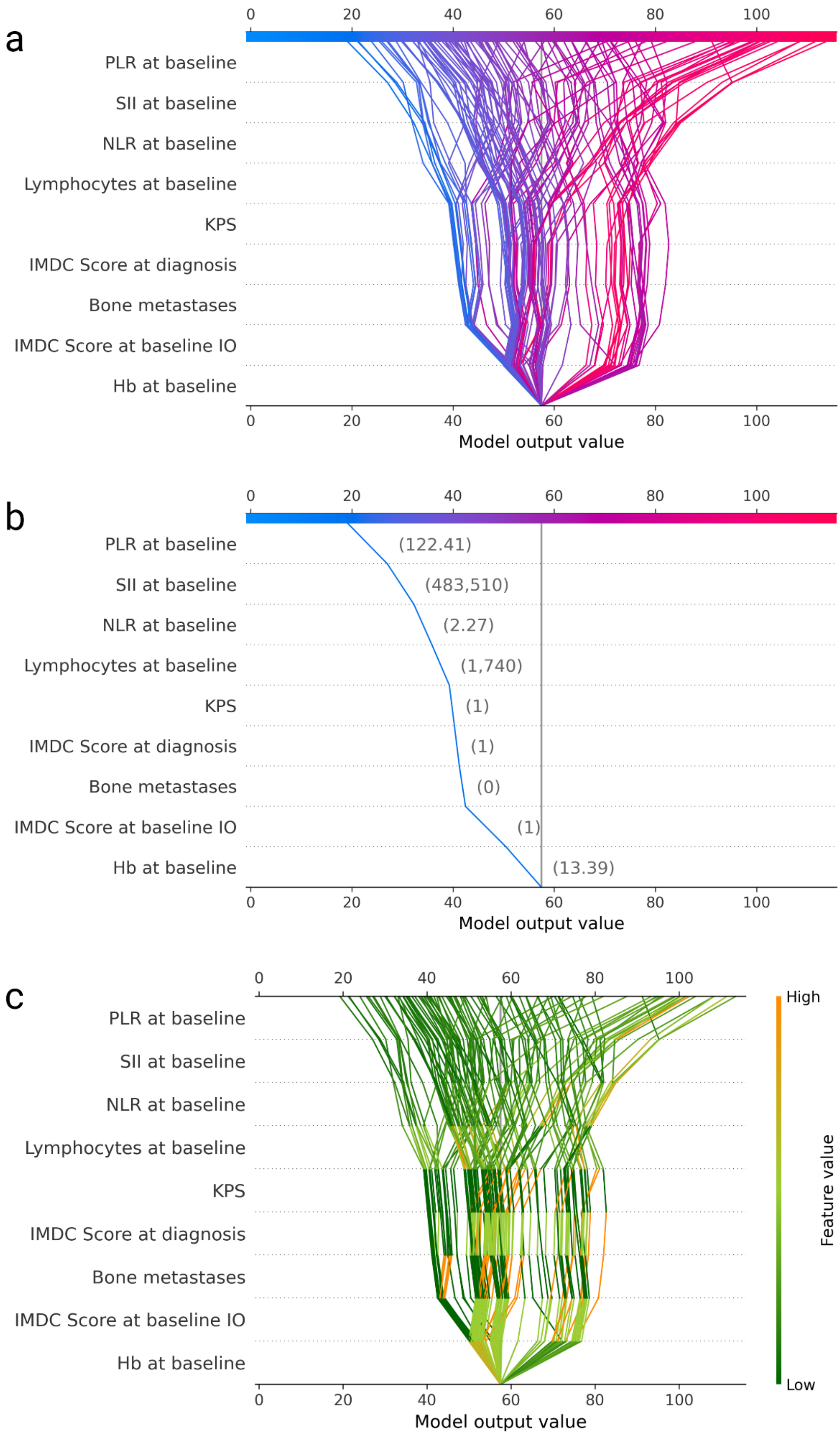

Supplementary Figure 12. Decision plots of RSF-univariate for OS on the test set, with features ordered by hierarchical clustering: (a) default plot; (b) patient predicted at the lowest risk; (c) color scaling related to feature values.

This visualization allows to see the model's functioning as a tree-like structure, showing that Hemoglobin splits the observations into groups, then the second feature from the bottom IMDC score strongly contributes to the prediction especially for the patients with high values for Hemoglobin at the baseline. Patients with high Hemoglobin and low IMDC score are shifted towards low risk.

STROBE Statement—Checklist of items that should be included in reports of ***cohort studies***

	Item No	Recommendation		Page No/section
**Title and abstract**	1	(*a*) Indicate the study's design with a commonly used term in the title or the abstract	✓	2
		(*b*) Provide in the abstract an informative and balanced summary of what was done and what was found	✓	2
**Introduction**				
Background/rationale	2	Explain the scientific background and rationale for the investigation being reported	✓	3
Objectives	3	State specific objectives, including any prespecified hypotheses	✓	3
**Methods**				
Study design	4	Present key elements of study design early in the paper	✓	4
Setting	5	Describe the setting, locations, and relevant dates, including periods of recruitment, exposure, follow-up, and data collection	✓	4
Participants	6	(*a*) Give the eligibility criteria, and the sources and methods of selection of participants. Describe methods of follow-up	✓	4-5
		(*b*) For matched studies, give matching criteria and number of exposed and unexposed		NA
Variables	7	Clearly define all outcomes, exposures, predictors, potential confounders, and effect modifiers. Give diagnostic criteria, if applicable	✓	5-7
Data sources/ measurement	8*	For each variable of interest, give sources of data and details of methods of assessment (measurement). Describe comparability of assessment methods if there is more than one group	✓	5-7
Bias	9	Describe any efforts to address potential sources of bias	✓	6-7
Study size	10	Explain how the study size was arrived at	✓	4
Quantitative variables	11	Explain how quantitative variables were handled in the analyses. If applicable, describe which groupings were chosen and why	✓	5
Statistical methods	12	(*a*) Describe all statistical methods, including those used to control for confounding	✓	5-7
		(*b*) Describe any methods used to examine subgroups and interactions	✓	5
		(*c*) Explain how missing data were addressed	✓	5-6
		(*d*) If applicable, explain how loss to follow-up was addressed	✗	NA
		(*e*) Describe any sensitivity analyses	✗	NA
**Results**				
Participants	13*	(a) Report numbers of individuals at each stage of study—eg numbers potentially eligible, examined for eligibility, confirmed eligible, included in the study, completing follow-up, and analysed	✗	NA
		(b) Give reasons for non-participation at each stage	✗	NA
		(c) Consider use of a flow diagram	✓	Fig. 1
Descriptive data	14*	(a) Give characteristics of study participants (eg demographic, clinical, social) and information on exposures and potential confounders	✓	Table 1
		(b) Indicate number of participants with missing data for each variable of interest	✓	Table 1
		(c) Summarise follow-up time (eg, average and total amount)	✗	NA
Outcome data	15*	Report numbers of outcome events or summary measures over time	✓	6, Suppl.

				data
Main results	16	(*a*) Give unadjusted estimates and, if applicable, confounder-adjusted estimates and their precision (eg, 95% confidence interval). Make clear which confounders were adjusted for and why they were included	✓	6
		(*b*) Report category boundaries when continuous variables were categorized	✗	NA
		(*c*) If relevant, consider translating estimates of relative risk into absolute risk for a meaningful time period	✗	NA
Other analyses	17	Report other analyses done—eg analyses of subgroups and interactions, and sensitivity analyses	✓	9-18; Suppl. data
**Discussion**				
Key results	18	Summarise key results with reference to study objectives	✓	18-19
Limitations	19	Discuss limitations of the study, taking into account sources of potential bias or imprecision. Discuss both direction and magnitude of any potential bias	✓	19-20
Interpretation	20	Give a cautious overall interpretation of results considering objectives, limitations, multiplicity of analyses, results from similar studies, and other relevant evidence	✓	20
Generalisability	21	Discuss the generalisability (external validity) of the study results	✓	19-20
**Other information**				
Funding	22	Give the source of funding and the role of the funders for the present study and, if applicable, for the original study on which the present article is based	✓	22

*Give information separately for exposed and unexposed groups.

Note: An Explanation and Elaboration article discusses each checklist item and gives methodological background and published examples of transparent reporting. The STROBE checklist is best used in conjunction with this article (freely available on the Web sites of PLoS Medicine at http://www.plosmedicine.org/, Annals of Internal Medicine at http://www.annals.org/, and Epidemiology at http://www.epidem.com/). Information on the STROBE Initiative is available at http://www.strobe-statement.org.
